# Forecasting the Effects of Global Change on a Bee Biodiversity Hotspot

**DOI:** 10.1002/ece3.70638

**Published:** 2024-11-29

**Authors:** Mark A. Buckner, Steven T. Hoge, Bryan N. Danforth

**Affiliations:** ^1^ Department of Entomology Cornell University Ithaca New York USA

**Keywords:** bee conservation, biodiversity, global change, joint species distribution model, protected areas, renewable energy

## Abstract

The Mojave and Sonoran Deserts, recognized as a global hotspot for bee biodiversity, are experiencing habitat degradation from urbanization, utility‐scale solar energy (USSE) development, and climate change. In this study, we evaluated the current and future distribution of bee diversity, assessed how protected areas safeguard bee species richness, and predicted how global change may affect bees across the region. Using Joint Species Distribution Models (JSDMs) of 148 bee species, we project changes in species distributions, occurrence area, and richness under four global change scenarios between 1971 and 2050. We evaluated the threat posed by USSE development and predicted how climate change will affect the suitability of protected areas for conservation. Our findings indicate that changes in temperature and precipitation do not uniformly affect bee richness. Lower elevation protected areas are projected to experience mean losses of up to 5.8 species, whereas protected areas at higher elevations and transition zones may gain up to 7.8 species. Areas prioritized for future USSE development have an average species richness of 4.2 species higher than the study area average, and lower priority “variance” areas have 8.2 more species. USSE zones are expected to experience declines of up to 8.0 species by 2050 due to climate change alone. Despite the importance of solitary bees for pollination, their diversity is often overlooked in land management decisions. Our results show the utility of JSDMs for leveraging existing collection records to ease the inclusion of data‐limited insect species in land management decision‐making.

## Graphical abstract

The American Desert Southwest is home to approximately one‐quarter of North American bee species. However, warming temperatures, shifting precipitation patterns, and development threaten to drive bee species redistribution, highlighting the need to include these data‐limited but ecologically vital species in land management decisions.

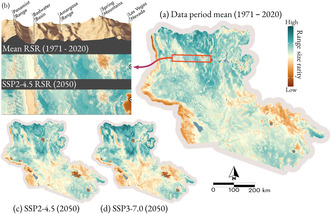

## 1 Introduction

We are experiencing the onset of the sixth mass extinction (Ceballos et al. 2015). The potential for biodiversity collapse threatens ecosystem health and human welfare, which is entwined with many of the same political, economic, and social practices driving the environmental crisis itself (Sage 2020). Humanity is responsible for the modification of 75% of the ice‐free land surface (Balvanera et al. 2019; IPCC 2022), the ballooning of atmospheric CO_2_ concentrations to levels not seen in the past two million years, and an increase in global terrestrial surface temperatures of 1.9°C over preindustrial averages (IPCC 2023a). These global changes drive species redistributions and habitat degradation, contributing to species declines, ecosystem simplification, and a reduced capacity to provide ecosystem services (Sage 2020).

Taking account of the looming crisis, many nations have adopted a new generation of biodiversity conservation initiatives built on the “30 by 30” framework, which seeks to protect 30% of the earth's land and ocean area by 2030 (Convention on Biological Diversity 2022; Dinerstein et al. 2019; European Commission and Directorate‐General for Environment 2021; Executive Order 14008 2021). The success of these initiatives depends on identifying and conserving areas of importance for biodiversity, ecosystem function, and ecosystem services. At present, over 1.2 million km^2^ are protected in the United States (US) alone (UNEP‐WCMC 2023), but these areas often have minimal utility for biodiversity conservation (Jenkins et al. 2015), instead prioritizing scenery in areas of otherwise limited economic value (Venter et al. 2018). While it is clear that we must emphasize biodiversity protection when establishing new protected areas, most assessments are taxonomically biased toward terrestrial vertebrates (Donaldson et al. 2017; Llorente‐Culebras, Ladle, and Santos 2023). Such a limited view of biodiversity is partly a result of deficient data for a substantial subset of species and ecosystems—a subset with a high proportion of threatened species (Borgelt et al. 2022).

Despite hosting relatively high biodiversity, deserts have historically received little conservation funding or research attention (Durant et al. 2012); now, these same ecosystems have been designated as priority areas for utility‐scale solar energy (USSE) development (Gasparatos et al. 2017). In the US, the Energy Act of 2020 increased the development pressure placed on the warm deserts of the southwestern United States by establishing a minimum goal of 25 gigawatts of permitted renewable energy development by 2025 on federal lands (National Goal for Renewable Energy Production on Federal Land 2020). While a rapid renewable energy transition is vital for addressing climate change, it may come at the cost of extensive land transformation (Capellán‐Pérez, de Castro, and Arto 2017). How this vast conversion of desert habitat for USSE will impact the ecosystem is unclear, particularly for insects (Grodsky, Campbell, and Hernandez 2021; Jeal et al. 2019), which provide vital ecosystem services but often have limited demographic, distribution, and habitat preference data. These constraints mean insects may receive little consideration in the establishment of new protected areas or land management decisions.

Bees, in particular, are of public and conservation interest for the pollination services they provide and recently documented declines (Biesmeijer et al. 2006; Burkle, Marlin, and Knight 2013; Powney et al. 2019; Turley et al. 2022). Home to upwards of a quarter of the approximately 4000 bee species in the US, the Desert Southwest contains remarkable bee species densities (Carril et al. 2018; Minckley and Radke 2021) making this region one of the most bee species‐rich in the world (Michener 1979; Orr et al. 2021). Many of the species documented from the southwest are host plant specialists, thought to be adapted to the variable and unpredictable desert environment (Minckley, Cane, and Kervin 2000), and may be facing the greatest risk of decline (Bartomeus et al. 2013; Biesmeijer et al. 2006; Bogusch, Bláhová, and Horák 2020; Wood, Holland, and Goulson 2016). While drivers of bee decline are numerous and spatially heterogeneous, many studies have focused primarily on pollinators in agroecosystems and the risks of pesticide exposure, which may be of lower importance in this region (Douglas et al. 2020). Instead, southwestern bee diversity is facing direct habitat loss from USSE, urbanization, and mineral extraction and indirect threats from introduced and invasive species (see Portman, Tepedino, and Tripodi 2019), and shifting disturbance regimes (Moloney et al. 2019).

The significance of climate change in bee declines remains debated (Dicks et al. 2021). As the southwest experiences more extreme temperatures (Garfin et al. 2018), increases in the duration and intensity of droughts (Williams et al. 2020), and changes in seasonal precipitation patterns (Abatzoglou and Kolden 2011), bees may need to modify their behavior, distribution, or development to respond to heat and water stress (Johnson et al. 2023). Shifting climate may also exacerbate habitat loss by altering ecosystem composition, degrading habitat quality, and reducing floral resource availability. For example, current warming and drought conditions have already been implicated in the reduction of desert vegetation (Hantson et al. 2021) and similar patterns are expected to continue, with forecasts predicting reductions in desert perennial and forb cover and changes to shrubland composition (Munson et al. 2012).

Here, we leverage publicly available bee collection records to map the spatial distribution of bee species diversity in the deserts of the American Southwest. We predict how bee species richness changes out to 2050, to explore how bee diversity may be affected by climate‐driven species redistribution and species declines. In addition, we focus on evaluating the suitability of existing protected areas and USSE development priority areas to understand the status of bee conservation and the potential threat posed by large‐scale land transformation for renewable energy development and urbanization. In doing so, we argue for the consideration of understudied and data‐deficient taxa in conservation and land‐use decision‐making through rapid and cost‐effective modeling approaches.

## 2 Materials and Methods

To explore how bee biodiversity might change under medium‐term (2041–2060) global change scenarios, we used projected land‐use change scenarios and climatology data consisting of an ensemble of eight general circulation models from the Coupled Model Intercomparison Project 6 (CMIP6) representing four shared socioeconomic pathways (SSPs; SSP1‐2.6, SSP2‐4.5, SSP3‐7.0, and SSP5‐8.5). SSPs represent greenhouse gas emissions, air pollution, and land‐use trajectories from varied global socioeconomic scenarios (Riahi et al. 2017). Each of these scenarios varies in the extent of projected change from net negative emissions by 2100 and global temperature change of < 2°C (SSP1‐2.6) to a further increase in emissions through 2100 (SSP5‐8.5; IPCC 2023b). We performed all analyses in R version 4.2.2 (R Core Team 2022).

### 2.1 Study Area

This study focused on the Mojave and Sonoran Basin and Range Omernik Level III ecoregions (Omernik 1987). In addition, we expanded the study area to incorporate the extent of the Desert Renewable Energy Conservation Plan (DRECP), which designates priority areas for USSE development in the California deserts (BLM 2016). To maximize the number of species that meet our minimum observation number (10 occurrences) for inclusion in our models and to reduce the risk of niche truncation by including a broader environmental gradient in our training data, we chose to buffer the study area boundary. We calculated the optimized buffer distance, *d*_*opt*_, by maximizing the log difference in species count per unit area as Equation (1):

(1) $$d_{\mathrm{opt}}=\underset{d\in D}{\arg\;\max}\left(\frac{\log\left(S_{d}-S_{1}\right)}{A_{d}}\right)$$

where *S*_*d*_ is the number of species in a region with area *A*_*d*_ associated with buffer distance *d* over a set of distances, *D*, ranging between 0 km and 48 km, and *S*_*1*_ is the species count in the unbuffered study area. Based on data availability, we only included bee records within the United States.

### 2.2 Bee Occurrence Data

We obtained occurrence records for all bee species in the Southwestern United States from the Global Biodiversity Information Facility (GBIF; GBIF.org 2023) and Symbiota Collections of Arthropods Network (SCAN; SCAN 2022). We removed duplicate records and identified several inconsistent near duplicates between the two datasets, which had identical metadata—including unique global identifiers—but different species names. Out of caution, we excluded all records from the US Department of Agriculture Bee Systematics Lab in Logan, Utah reported in SCAN, which we found contained incorrect species names for some records. In addition, we removed iNaturalist observations entered in SCAN which reported both withdrawn identifications and currently accepted identifications for the same observations as separate records. Given these discrepancies, we prioritized GBIF records which reported species names that were consistent with the original datasets and known from the area.

For this study, we selected a 1 km grain to represent the spatial scale at which most bees interact with their environment (Kendall et al. 2022). We acknowledge that unreported or uncertain location data derived by geolocating collection records are common and our chosen grain may be smaller than the uncertainties of some records. This “positional certainty‐ecologically informative grain” trade‐off was explored in a recent analysis which suggested that although uncertainty can reduce model performance, a coarser grain, beyond the scale at which the organism interacts with its environment, may be more detrimental (Gábor et al. 2022). To reduce uncertainty from georeferencing issues, we excluded records with a reported uncertainty > 5 km and checked for localities corresponding to capitals, administrative area centroids, or biodiversity institutions with coordinatecleaner (ver. 2.0–20; Zizka et al. 2019). We compiled the final dataset by excluding any records that fell outside the buffered study area, were collected before 1971 or after 2020, had coordinates within water bodies, or represented species with < 10 unique records.

To assess if there are any differences between how life history groups respond to climate change and urbanization, we accumulated trait data for each species regarding the degree of host plant specialization, nesting behavior (e.g., above, or below ground nest construction), life stage during winter diapause, and life history (social, solitary, or parasitic). We classified bees as host plant specialists when they were only known to collect pollen from plants within a single family or, if they were brood parasites, they parasitize a species classified as a host plant specialist (Table S1).

### 2.3 Pseudo‐Absence Points

The available bee occurrence records in the region are the result of opportunistic surveys without absence data and are biased toward human population centers, roadways, and certain protected areas. To facilitate modeling, we used a two‐stage approach for pseudo‐absence point selection. For clarity, we will distinguish between “presence‐background” and “pseudo‐absence” methods, though these terms are often used interchangeably (Sillero and Barbosa 2021). Here, we characterize presence‐background methods as those that use many random or biased random points to represent the full range of environmental conditions available to the species being modeled (Phillips et al. 2009). We contrast this with pseudo‐absence methods which seek to generate artificial absence data (Sillero and Barbosa 2021).

First, we calculated a normalized kernel smoothed intensity function using all available occurrence records with a 30 km bandwidth to generate a bias layer (spatstat ver. 3.0–6; Baddeley, Rubak, and Turner 2015). The resulting normalized values were treated as weights to generate 10,000 background points across the study area with similar spatial bias as the presence data (Inman et al. 2021; Kujala, Whitehead, and Wintle 2015; Valavi et al. 2021). We used this initial set of background points to train balanced random forest models (BRF) for each species independently. BRF addresses class imbalance in presence‐background datasets to improve predictive performance by subsampling the majority class (the background points) to equal the minority class (the presence points) at the level of each tree (Valavi et al. 2021). We trained BFR probability trees with the default parameters to estimate the occurrence probabilities of each bee species (ranger ver. 0.14.1; Valavi et al. 2021; Wright and Ziegler 2017). To further refine the models, we applied recursive feature elimination, where environmental covariates were excluded based on the mean variable importance across all species. We retained the simplified models if removing a given covariate increased the area under the receiver operating characteristic (ROC) curve (AUC) for at least half of the species‐level models. During each step, we estimated model performance with 5‐fold spatial block cross‐validation implemented with blockcv (ver. 3.0–0, Valavi et al. 2019).

In the second stage, we sampled occurrence localities represented in our dataset independently for each species to use as pseudo‐absence points. We weighed each record with the complement of the mean predicted species‐wise occurrence probabilities from the BRF models. We chose to limit the number of background points to equal the number of presence points for each species or a maximum of 200 for honey bees ( *Apis mellifera* Linnaeus 1758)—a widespread species that may not be regularly collected or reported during opportunistic surveys, even if it is observed.

Our method of pseudo‐absence point selection allowed us to overcome several limitations of current Joint Species Distribution Model (JSDM) implementations. Notably, nearly all JSDMs require presence–absence or abundance datasets—with some exceptions in development (see Deneu et al. 2021). In presence‐background methods, the number of background points should represent the full extent of available environmental conditions (Phillips et al. 2009). However, due to the considerable number of points required to do so, presence‐background approaches are unfeasible, in part, because the computational cost of JSDMs scale poorly with data size (Ovaskainen and Abrego 2020). By selecting the presence localities of other species as pseudo‐absence points, we can avoid increasing the size of the dataset significantly while also ensuring that the pseudo‐absence points are well informed and have similar bias as the presence points. In previous studies, related multistep approaches outperformed random background points by directly accounting for spatial variation in sampling bias and environmental associations (Iturbide et al. 2015; Senay, Worner, and Ikeda 2013). The method we present here extends this concept from these multistep approaches to modeling contexts which scale poorly with large numbers of background points.

### 2.4 Environmental Data

We considered a maximum of 18 possible environmental covariates that may affect bee distribution and habitat suitability (Table S1). Broadly, these variables represent climate (temperature and seasonal precipitation), soil sand and clay content, topography, urban land use, and forested and unforested land cover. We reprojected all environmental covariates to a custom WGS84 Albers Equal Area Conic coordinate system and resampled to 1 km resolution. Climate data were obtained from climatena (ver. 7.31), an application that downscales gridded climate data from a 4 km grid to a user‐specified grain through dynamic local downscaling (Mahony et al. 2022; Wang et al. 2016). We acquired climate layers representing the average climatic conditions for each decade between 1971 and 2020 and calculated the future climate as the 10‐year average of the projected annual data from climatena for 2021–2030, 2031–2040, and 2041–2050 for all four SSPs. Given the medium‐term time period, we assumed that the topographic and soil composition covariates will remain stable. To limit the effect of correlation on the transferability of our models, we identified all variables with an absolute Pearson's correlation coefficient > 0.7 and excluded correlated variables which we expect to have lower importance for bee distributions (Feng et al. 2015). To identify any shifts in correlation structure, we checked for changes in correlation coefficients between the data period (1970–2020) and the future period (2021–2050) greater than ±0.1. We evaluated the extent to which extrapolation may influence our predictions under future conditions by calculating Multivariate Environmental Similarity Surfaces (MESS) for each future decade with modeva (ver. 3.13.3, Elith, Kearney, and Phillips 2010; Barbosa et al. 2013).

### 2.5 Joint Species Distribution Model

Obtaining sufficient records to meet the suggested minimum sample sizes for single species distribution models may be impractical in community wide studies, where rare or threatened species are often a priority. To overcome this limitation, we trained JSDMs through the Hierarchical Modeling of Species Communities (HMSC) R package (hmsc ver. 3.0–14, Tikhonov et al. 2023). As opposed to stacked species distribution models, which assume each species responds independently to its environment, JSDMs assume a joint response (Norberg et al. 2019). By modeling all the species together, HMSC allows for species–environment relationships to be refined through species associations and residual variation allowing rare species to “borrow strength” from more common species (Norberg et al. 2019).

We ran four different JSDM models of varying complexity, (i) a spatial random effect only, (ii) climate covariates with no spatial random effect, (iii) all topographic and climate covariates with no spatial random effect, and (iv) all topographic and climate covariates with a spatial random effect. We used the default priors for each model and the spatial models were fit using the nearest neighbor Gaussian process (Tikhonov et al. 2020). While phylogenetic and trait data can be informative for these models, incomplete data and limited natural history observations prevented us from applying it to this analysis. For each model, we drew 500 samples from four Markov Chain Monte Carlo chains for a total of 2000 samples. Each chain consisted of a 62,500‐iteration transient period followed by sampling with a thinning interval of 250 iterations for a total of 187,500 iterations per chain. We assessed model convergence using potential scale reduction factor (Gelman and Rubin 1992). Finally, we evaluated the explanatory power of the models with AUC and Continuous Boyce Index (CBI), a threshold independent measure model performance with presence‐only data (Hirzel et al. 2006). Due to data limitations, we evaluated our models predictive performance when extrapolating into novel environments with two fold spatially blocked cross‐validation (Roberts et al. 2017; Valavi et al. 2019).

### 2.6 Climate Change and Land Use

To evaluate the impacts of climate change and urbanization, we predicted the probability of occurrence for each grid cell using future climate and urbanization data for each decade and scenario. We explored the change in predicted bee richness across the study area and calculated the marginal effect of distance to the closest natural area (i.e., non‐urban land cover) from the posterior sample to evaluate how the bee assemblage responds to urban sprawl. For each species, we calculated the current and future occupied area as the sum of occurrence probabilities within the study area to avoid biases from thresholding the SDM predictions (Stark and Fridley 2022). We then calculated range‐size rarity (RSR) as a metric of grid cell importance for bee conservation in the region by identifying cells that contribute the most to the total occurrence area of range‐restricted species (Guerin and Lowe 2015). To calculate RSR without applying a threshold, we took the sum of each species occurrence probability divided by their occurrence area in each grid cell.

We evaluated how well existing protected areas overlap with predicted bee diversity by calculating the proportion of each bee's total occurrence area within protected areas. To evaluate if protected areas differ in suitability from equivalent unprotected areas, we used the marginal effects of protected area status where all other covariates were set equal to their mean (Bakx et al. 2023). Additionally, we evaluated how the utility of individual units varies across the study area by calculating the change in average richness for each individual protected area. For this analysis, we only considered protected areas that are actively managed for conservation as reported by the US Geological Survey Protected Areas Database (USGS GAP 2022).

Finally, we assessed how USSE development may affect bee diversity within the Southwestern United States by exploring predicted species richness in areas prioritized for solar development as part of the 2012 Programmatic Environmental Impact Statement for Solar Energy Development (Solar PEIS; BLM and DOE 2012), 2013 Restoration Design Energy Project (RDEP; BLM 2013), and 2016 Desert Renewable Energy Conservation Plan (DRECP; BLM 2016). We evaluated whether these current plans prioritize solar development in areas of high bee diversity and whether these areas may be of high conservation value in the future by calculating the change in average and per unit richness in each decade and climate change scenario. Additionally, we calculated the proportion of each species occurrence area within solar development areas to identify whether certain species faced a risk substantial habitat loss from USSE development.

## 3 Results

**FIGURE 1:**
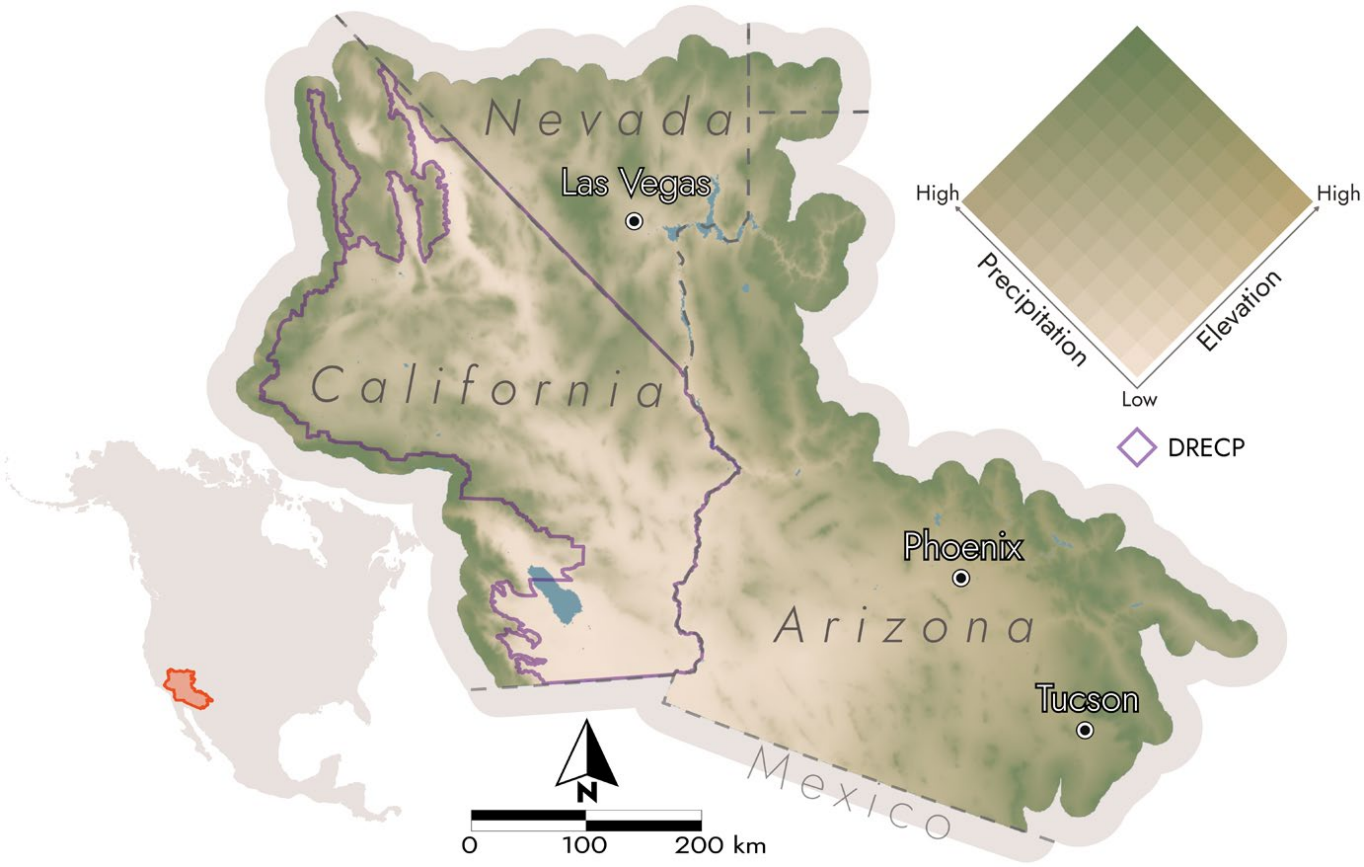
The study area covers the Mojave and Sonoran Ecoregions and the Desert Renewable Energy Conservation Plan (DRECP) area (purple) across portions of four states in the Southwestern United States. The terrain color ramp corresponds to precipitation and elevation. The low, dry desert basins and valleys are colored tan, and the comparably wet mountains, plateaus, and uplands are green.

Our integrated and cleaned bee occurrence dataset included 5731 records over the 5‐decade data period between 1971 and 2020, where each record represents the collection of a species within a grid cell during a certain decade (see Supplemental Code). Before buffering the study area, we identified 131 species that met the minimum requirements for modeling. After buffering the study area boundary by 12 km, an additional 17 species met the modeling requirements with a median of 22 unique location‐decade records each bringing the total number of modeled species to 148. Apart from honey bees (*n* = 1163), no species had more than 200 unique records. The final study area covers approximately 300,000 km^2^, including portions of Arizona (AZ), California (CA), Nevada (NV), and Utah, and spans over 4000 m in elevation (Figure 1).

Out of our original 18 covariates, many temperature variables, including growing degree days, frost free period, and minimum temperatures, were highly correlated with extreme maximum temperature. The shift in correlation between the data period and future climate data were < 0.1 for all pairs of covariates except for extreme maximum temperature and summer precipitation in 2050 under SSP2‐4.5 which shifted by 0.12. The final covariates we chose to include in the model were extreme maximum temperature, summer and winter precipitation, annual temperature range (Wang et al. 2016), distance to natural areas within urbanized land use (Chen, Li, and Liu 2022; ESA 2017), eastness, northness, terrain ruggedness index (Amatulli et al. 2020), soil sand content (Hengl 2018), and protected area status (USGS GAP 2022).

### 3.1 Joint Species Distribution Models

We explored fitting JSDMs with a spatial random effect; however, despite recent improvements showing promise for more efficient fitting of large spatial models (Tikhonov et al. 2020), we were not able to achieve convergence due to long computational times. Two of the four models, climate covariates only and all environmental covariates, both without spatial random effects, reached satisfactory convergence with potential scale reduction factors < 1.1 for all parameters. Of the converged models, the simplest model performed worse than the more complex model that included both climate and topographic covariates in both explanatory and predictive performance. Overall, the more complex model performs satisfactorily in explanatory tasks with an average AUC of 0.86 (range: 0.65–1.00) and CBI of 0.67 (range: −0.50 to 0.98). As expected, the predictive performance was lower on average, with a mean AUC of 0.67 (range: 0.05–1.00) and CBI of 0.38 (range: −0.95 to 0.96). We performed all the following analyses using the model with all environmental covariates and no spatial random effect.

**FIGURE 2:**
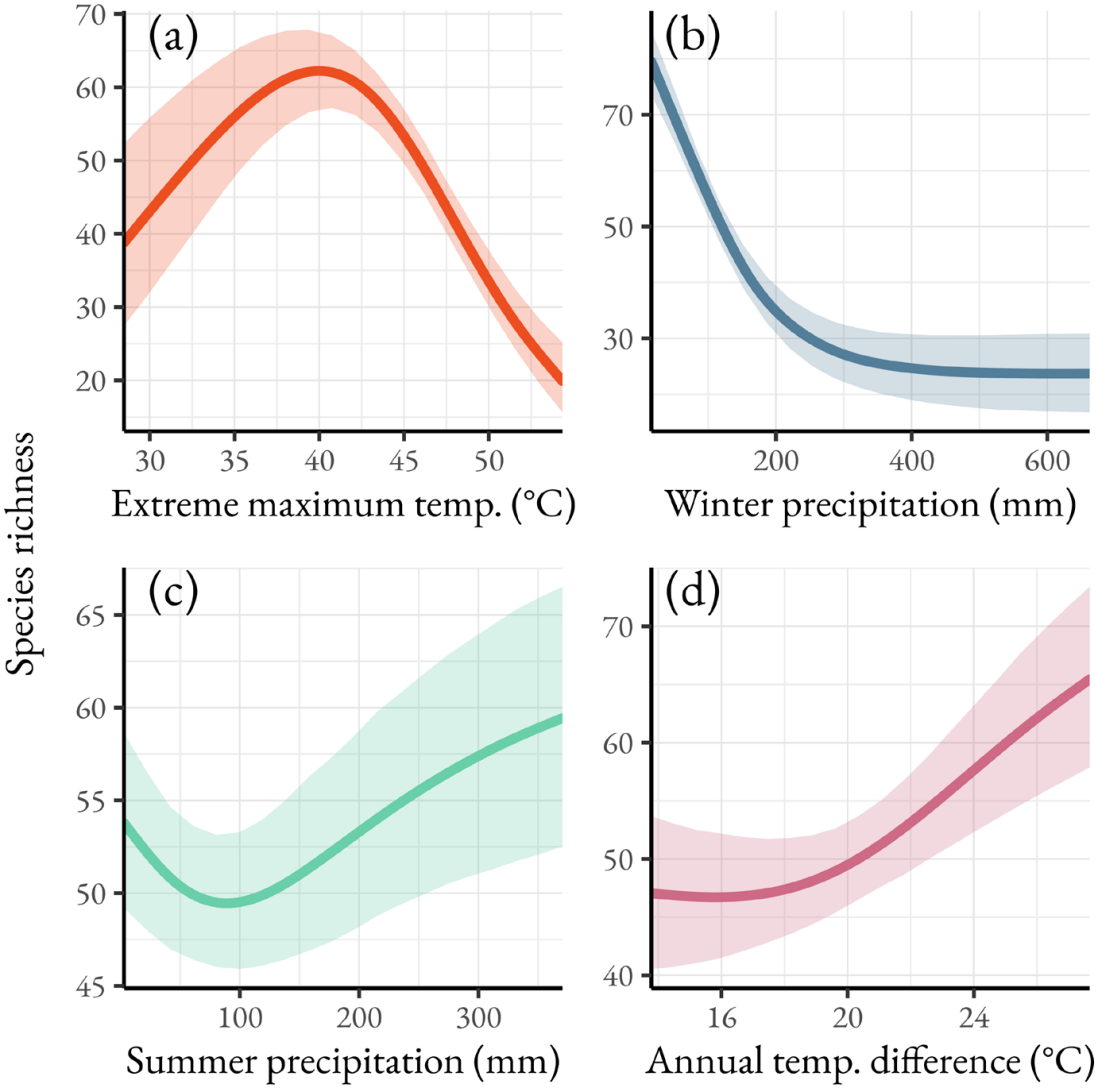
Species richness response curves to (a) extreme maximum temperature, (b) winter precipitation, (c) summer precipitation, and (d) annual temperature difference, the difference between the mean temperature during the warmest and coldest months. The shaded regions represent the 95% credible intervals.

The models suggest that climate and urbanization are the most important predictors of bee occurrence in the region. Winter precipitation accounted for a total of 24.2% of the explained variance on average, followed by distance to natural land cover with urban areas (21.5%), extreme maximum temperature (15.0%), and summer precipitation (11.5%). The remaining covariates individually accounted for no more than 7.5% of the explained variance but accounted for a substantial combined total of 27.8%. The model predicts species richness is highest in regions with moderately high extreme temperatures, peaking around 40°C and declining markedly at temperatures above this peak (Figure 2a). The effects of precipitation varied depending on the season, where higher winter precipitation resulted in lower species richness (Figure 2b). In comparison, species richness across the gradient of summer precipitation was more stable, with minor increases in richness with higher precipitation (Figure 2c). Additionally, modeled richness increased in regions with a greater difference in mean temperature between the warmest and coldest months (Figure 2d).

### 3.2 Climate Change and Urbanization

Overall, 70% (SSP2‐4.5) to 90% (SSP3‐7.0) of the study area experienced a loss of species richness by 2050, calculated as the proportion of grid cells with declining species richness under each climate change scenario. Species occurrence areas declined by an average of 29 km^2^ (18.5%) between 1980 and 2020. Apart from SSP 3–7.0 which continued to decline by 1.6 km^2^, our model predicts the average occurrence area will increase slightly between 2020 and 2050 by 4 km^2^ to 7.3 km^2^. The extent of habitat loss varied minimally between species grouped by life history traits. The greatest difference occurred between generalists and specialists, with generalists experiencing an average loss of only 6.5 km^2^ more than specialists. Between 23% (SSP3‐7.0) and 43% (SSP2‐4.5) of species are predicted to expand their ranges under climate change, with the most notable being introduced and managed honey bees. We did not find evidence of novel environmental conditions under future conditions, except for distance to natural areas, which required extrapolation with distances > 8 km (Figure S1).

**FIGURE 3:**
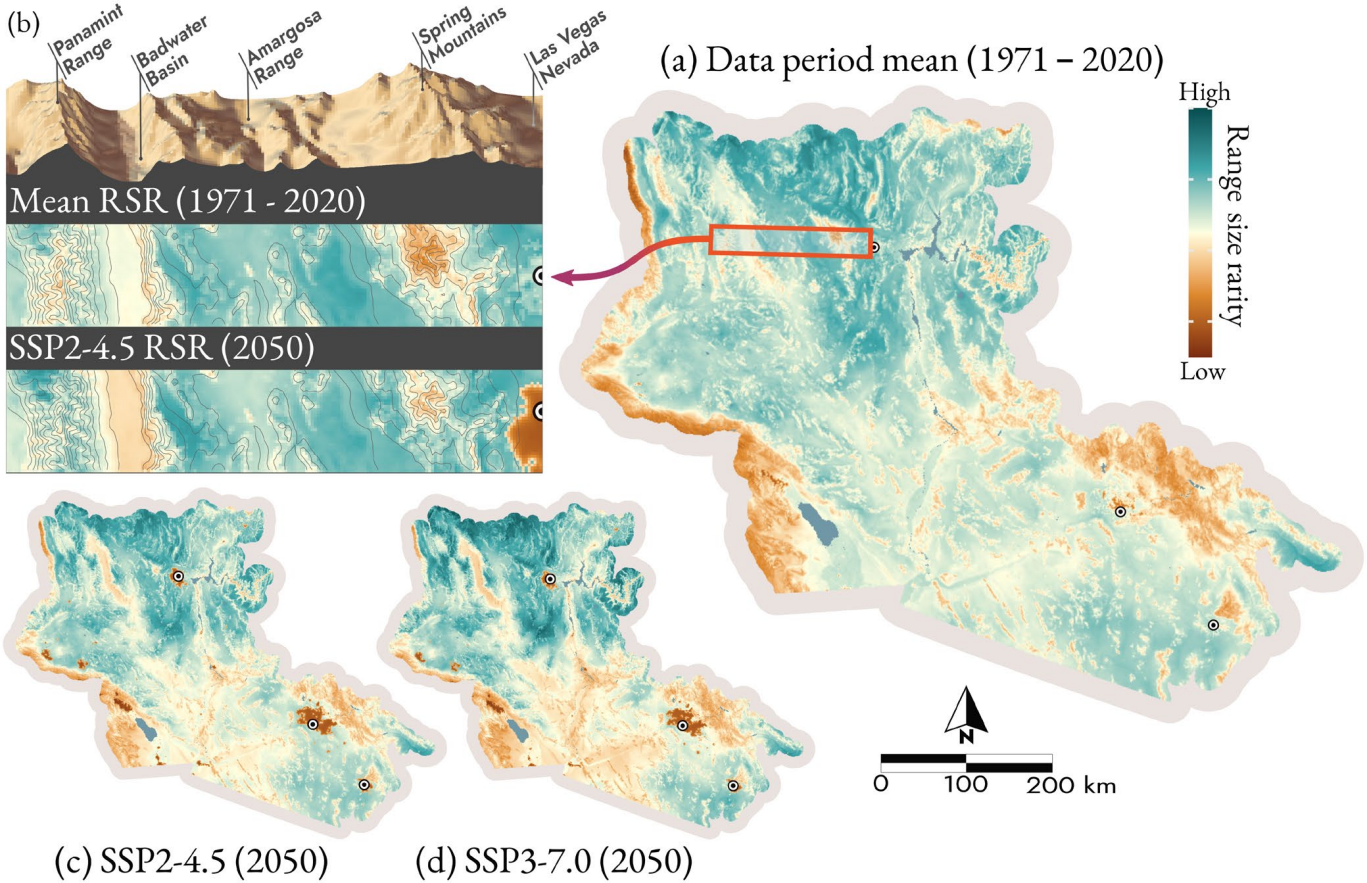
RSR is heterogeneous across the landscape (a), decreasing in low‐lying valleys and urbanized areas and increasing at mid‐elevations under future climate and land‐use predictions (b). RSR decreases in the Sonoran between (a) the data period, calculated as the mean of five decades between 1971 and 2020, and 2050 under (c) SSP 2–4.5 and (d) SSP 3–7.0. The major metropolitan areas indicated with points are, from north to south, Las Vegas, NV; Phoenix, AZ; and Tucson, AZ.

During the data period, low to mid‐elevations—with an exception for the greatest extremes such as Death Valley, CA—are the most important for range‐restricted species (Figure 3a). RSR, the relative grid cell importance for range‐restricted species, declined in the lower Sonoran under future predictions and increased in the mountainous areas in the Mojave and valleys at higher latitudes and elevations, for example, the Spring Mountains, NV, and Arizona Upland (Figure 3b). Nevertheless, the general pattern across the region remained similar to the data period, with the regions of greatest importance localized to the lower‐lying areas of the Mojave and the eastern extent of the Sonoran Desert (Figure 3c,d). Beyond climate change, urbanization showed a marked effect on species richness and RSR. Within urban areas, richness declined in urban land uses as the distance to natural areas increased (Figure S2). Bees with different nesting behaviors responded differently, with the total proportion of species that nest above ground increasing by up to 1.8% in urban areas, reaching the greatest proportion of above ground nesting species at about 2.5 km from natural land cover.

### 3.3 Protected Areas

**FIGURE 4:**
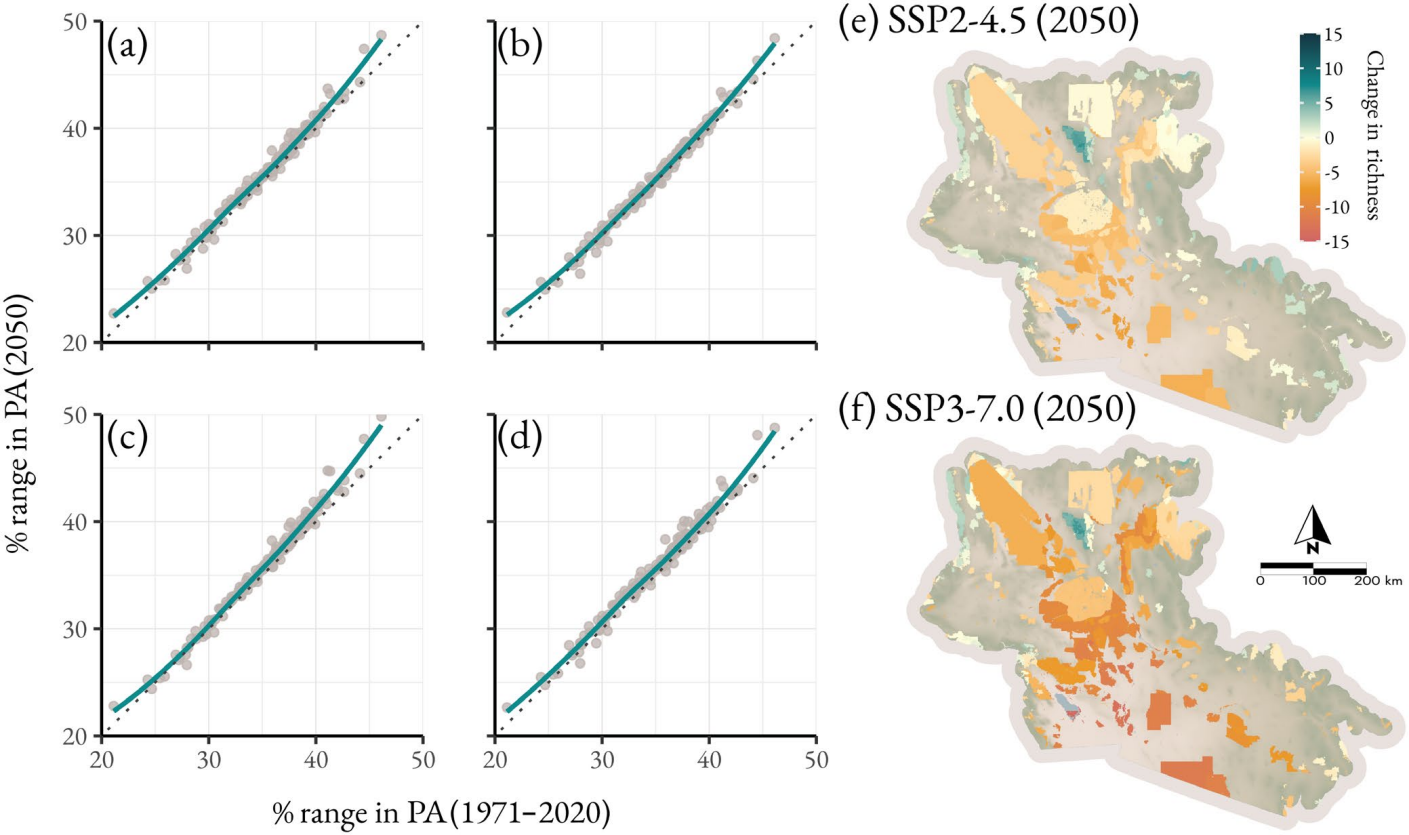
Comparison between the percent of each species occurrence area in protected areas in the data period and 2050 under (a) SSP1‐2.6, (b) SSP2‐4.5, (c) SSP3‐7.0, and (d) SSP5‐8.5. The points represent individual bee species, and the overall trend is shown with a smoothed line and dotted 1:1 line. Panels (e) and (f) show the change in per unit richness between the 1971–2020 mean and 2050 under (e) SSP2‐4.5 and (f) SSP3‐7.0.

Existing protected areas contained an average of between 21.1% and 46.1% of the predicted occurrence area for each species within the study area during the data period. The proportion of each species occurrence area contained within protected areas changed by between −1.5% for *Halictus tripartitus* (Cockerell 1895) to 3.7% for *Hoplitis producta* (Cresson 1864) between the data period and 2050 under any SSP (Figure 4). The species with the greatest proportion of their occurrence area within protected areas have the highest occurrence probabilities at higher elevations and in the Mojave (e.g., *H. producta* and *Megandrena enceliae* Cockerell 1927), and the lowest are found primarily in the eastern Sonoran and valley floors (e.g., *Xylocopa sonorina* Smith 1874 and *Epimelissodes duplocincta* Cockerell 1905). The marginal effects of protected areas suggest land managed under conservation mandates is no more or less suitable for any species when all other covariates are set to their mean (89% credible interval). Species richness in protected areas is predicted to decline compared to the data period average in all SSPs, with SSP 3–7.0 resulting in the greatest loss (mean = −5.8 species) and SSP 2–4.5 the lowest (mean = −2.2 species). Predicted declines vary widely across individual protected area units. By 2050, units proximal to urban areas in the Coachella Valley, CA, experience the greatest declines, losing between −27.4 species (SSP 3–7.0) and −43.8 species (SSP 5–8.5). Whereas units found within mountainous areas in and around the Mojave and the Arizona Upland increase in mean richness by up to 6.6 species in SSP 1–2.6 or 7.8 species in SSP 3–7.0 by 2050 (Figure 4). Overall, a minimum of 72% of protected land area may experience declining bee richness by 2050 with intermediate climate change (SSP 2–4.5), reaching up to 86% under SSP 3–7.0.

### 3.4 USSE Development

**FIGURE 5:**
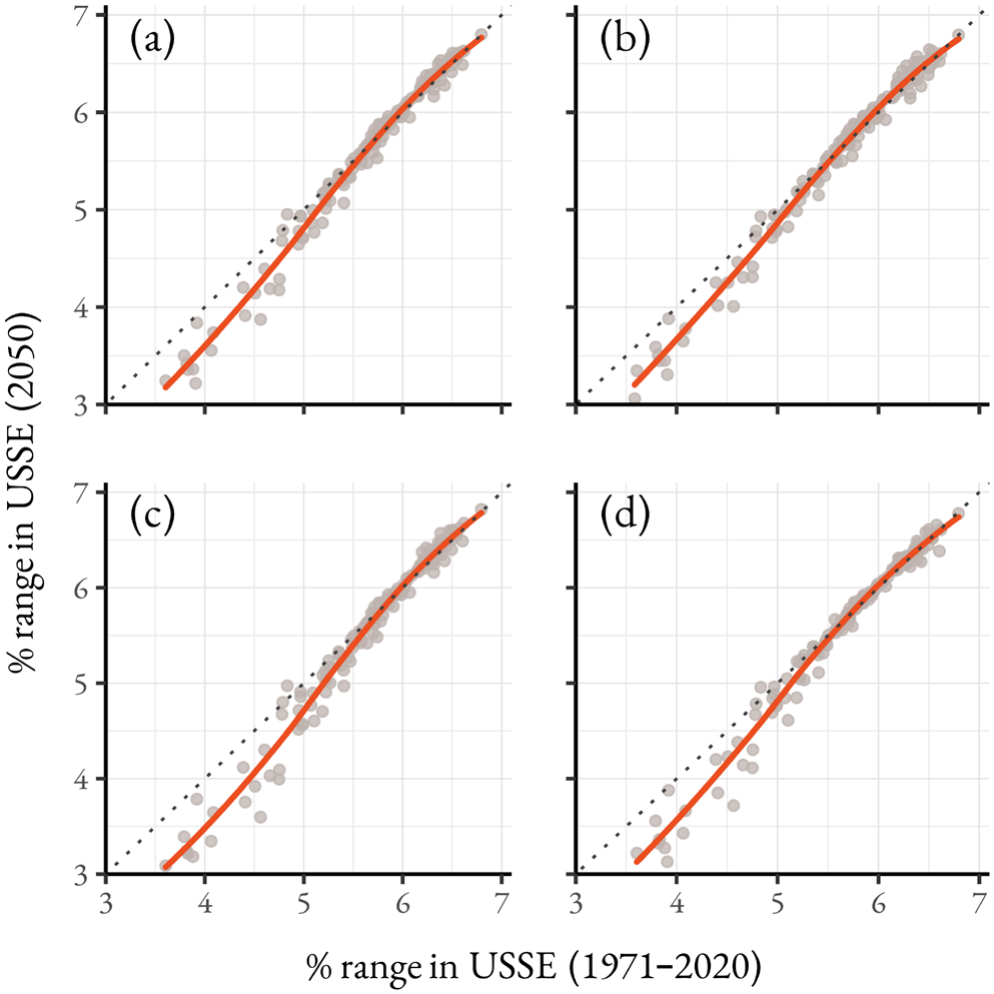
Comparison between the percent of each species range in solar energy development areas during the data period (1971–2020) and 2050 under (a) SSP1‐2.6, (b) SSP2‐4.5, (c) SSP3‐7.0, and (d) SSP5‐8.5. The points represent individual species, and the deviation of the trend from the dotted 1:1 line is shown with a smoothed line.

Utility‐scale solar energy priority and variance areas, which span over 16,000 km^2^ in the study area, contained above average species richness during the data period (priority areas: mean = 74.2 species, variance areas: mean = 78.2 species, study area: mean = 70.0 species). By 2020, the predicted richness of these areas declined to an average of 65.1 species in priority areas (variance areas: 71.8 species) after which predicted species richness did not continue to decline, remaining between 63.6 (SSP 3–7.0) and 68.4 species (SSP 5–8.5) on average. The individual responses of species vary depending on the extent to which their range overlapped with USSE development areas during the data period. For bees with the greatest proportions of their range in USSE development areas, the predicted occurrence area overlapping with USSE does not deviate substantially from the data period average. In contrast, species with < 5.5% of their occurrence area in USSE development areas had a smaller overlap under future conditions (Figure 5). Overall, the average richness of USSE development areas is expected to decline by 2050 in all climate scenarios with the largest declines in SSP 3–7.0 (−8.0 species) with all other scenarios declining by between −2.7 (SSP 2–4.5) and −4.7 (SSP 1–2.6) species. Like protected areas, the greatest declines are expected in USSE units near urbanizing land uses with a maximum loss between −59.4 species in SSP 3–7.0 and −63.1 species in SSP 5–8.5. Notably, some units within transition zones may see an increase in richness between 4.8 species (SSP 1–2.6) and 5.3 species (SSP 2–4.5).

## 4 Discussion

Our findings suggest that the diverse arid‐adapted bee assemblage of the Mojave and Sonoran deserts is not immune to the effects of climate and land‐use change. By accounting for the changing climate and extensive urbanization, our model suggests that bee richness is likely to decline across up to 90% of the Mojave and Sonoran ecoregions land area under midterm (2041–2060) global change, although the effects are heterogeneous across the region and climate change scenario. Current protected areas are not uniquely suited for protecting bee diversity compared to surrounding unprotected land, and on the whole existing units will continue to protect a substantial portion, at least 21%, of both current and future bee ranges, even though we expect richness to decline in many individual units.

Our model supports the assertion that warming temperatures and altered precipitation patterns will negatively affect native bee diversity in the coming decades. In the context of the few existing arid‐adapted bee species distribution models, our findings, with few exceptions (e.g., honey bees), differ in predicting range contractions as opposed to expansions (Dew, Silva, and Rehan 2019; Silva et al. 2018). Although the implications of climate change remain uncertain, explorations of thermal physiology suggest that desert bees may be vulnerable to extreme heat and desiccation, although the effects vary depending on the species and past exposure (Barrett et al. 2022; Bennett et al. 2021; Burdine and McCluney 2019; Chappell 1982; Hamblin et al. 2017; Johnson, Alvarez, and Harrison 2023). In this study, bee richness peaked at an intermediate extreme temperature of around 40°C, suggesting extreme high temperatures reduce habitat suitability for many species. Even so, bees may be able to respond through altered behavior or adaptation (Johnson et al. 2023). Bet hedging, in particular, could help ensure bee emergence remains synchronous with host plant bloom year to year and the most hazardous environmental conditions are avoided (Danforth 1999). Such adaptations may prove particularly important for persistence under future climate conditions, as increasingly long duration droughts make floral resource availability more unpredictable (Minckley, Roulston, and Williams 2013).

Beyond climate change, one of the most prominent examples of land‐use change driving habitat loss in the region is the expansion of rapidly growing urban areas (Wu et al. 2011). Our models predicted that continued urban sprawl, and concurrently the infilling of habitat fragments in desert metropolises (Shrestha et al. 2012), is likely to exacerbate bee declines. Existing literature on the effects of urbanization on desert bees supports our findings. Previous work in Phoenix, AZ, found that bee richness is higher in desert habitats outside urban areas than in urban desert fragments or residential areas (Hostetler and McIntyre 2001). In addition, our findings of a minor increase in the proportion of cavity nesting bees in urbanized areas are consistent with past observations in the urban matrix of Tucson, AZ. Here, Cane et al. (2006) found an increase in nesting resource availability for cavity nesting bees resulted in an overrepresentation of these species in urban fragments relative to undeveloped desert sites.

Within the near term, USSE is poised to contribute to rapid and extensive land‐use change in the southwest. While the exact implications of solar installations on insects are not fully known, our results indicate that development is slated to occur in regions of high bee diversity. The degree to which solar installations may contribute to additional declines in bee richness will likely depend on the method of site preparation. For non‐bee floral visitors, any method of site preparation, whether blading or mowing, resulted in displacement (Grodsky, Campbell, and Hernandez 2021) and both methods reduce perennial plant and cacti cover, potentially limiting floral resource availability (Grodsky and Hernandez 2020). While we were not able to directly model the effects of solar development, our results suggest that USSE priority areas contain high bee richness under both current and future climatic conditions. Even so, development is not occurring in climate refugia, and we predict developing areas to experience declining richness and lower importance for range‐restricted species in the future due to climate change, particularly in the Sonoran Desert. We note some exceptions to this trend in units within the Arizona Upland and northern Mojave. Individual species may have different degrees of susceptibility to the co‐occurring threats of development and climate change, and we emphasize that unraveling the consequences of these threats happening concurrently is a critical area of future research required to rigorously evaluate the risks posed by USSE to pollinators.

While urbanization and renewable energy development are expanding, much of the Desert Southwest is composed of protected lands that are actively managed for conservation. These protected areas currently overlap with a minimum of 21% of each bee species occurrence area, but not all units maintain high richness under midterm predictions with over 72% of protected land area expected to experience declines. As with bee richness overall, protected areas in the Sonoran Desert and low‐lying areas in the Mojave are likely to experience declining richness in the future. Units located nearest to cities showed the greatest loss of species richness in our models, but it is reasonable to equate these to large desert fragments which are still able to maintain relatively high levels of bee diversity compared to the surrounding urban matrix (Cane et al. 2006), suggesting that our model may be overpredicting species losses in this circumstance. Our results suggest an increasing importance of conserving habitats at higher elevations in the Mojave and the Arizona Uplands. The declining richness of protected areas highlights the need to reconsider establishing new conservation lands to protect climate refugia. As bees redistribute to track their climate niche, higher elevations and topographically rough landscapes could provide suitable microclimates for displaced species. Central to the concept of “conserving nature's stage” (Beier, Hunter, and Anderson 2015), prioritizing abiotic diversity may serve to better protect southwest pollinators in the coming decades.

Our models were not able to account for the full complexity of species‐ecosystem interactions under global change. Various aspects of how bees respond to climate and land‐use change cannot be readily accounted for in correlative presence/pseudo‐absence models, such as host plant interactions, dispersal ability, or demographics. Moreover, our models may not account for behavioral or adaptive responses to the environment which may make species less susceptible to climate change. Such uncertainty is particularly evident in situations where the future environmental conditions do not have a present‐day analog requiring extrapolation into novel conditions, which we only identified with extreme distances to natural areas under future urban sprawl. We further caution that our predictions represent our best hypothesis of how future change may impact richness in the region. The usefulness of AUC for presence/pseudo‐absence data is limited (Golicher et al. 2012); however, it remains the most common method to measure the support of presence‐only models (Konowalik and Nosol 2021). CBI, a threshold independent metric for presence‐only model performance, showed similar results to AUC with the model on average preforming well in explanatory tasks but only moderate performance when transferred across space. Lastly, it is important to acknowledge that the use of pseudo‐absence data itself required assumptions about where each species is least likely to occur which may introduce uncertainty in the species–environment response and stochasticity in the predictions themselves. Ideally, such an analysis could be replicated many times and the results combined to generate final predictions; however, the long runtime of our models made this impractical. Null model approaches that require repeat model fitting using random draws of presence records are similarly impractical (Raes and ter Steege 2007). Our approach using existing occurrence locations of other species means that both pseudo‐absence and presence data may not represent the full environmental response for each species. While we used the best available data, future work in this area would benefit from additional records, ideally structured collection efforts that aim to reduce collection biases, report absence localities or non‐detections, and better represent the full environmental gradient in the region.

Considering the rapid pace of development and push for the establishment of new biodiversity targets, embracing modeling methods is necessary to facilitate the consideration of invertebrate species in conservation decision‐making. While the push for a structured national pollinator monitoring program is gaining ground (Woodard et al. 2020) many of the threats to pollinator diversity are allowed to move forward, as land managers and policymakers are unable to adequately consider the potential impacts due to deficient historical data. The DRECP exemplifies this need, in that the planners acknowledged the high diversity of invertebrate species within the planning area but excluded them from consideration in the planning process (BLM 2016). The work we present here serves as an example of leveraging existing data sources to predict the distributions of rare and data‐limited species. We emphasize the need to consider future climatic conditions in the establishment of protected areas and new development. Conservation actions, such as increasing native vegetation in residential areas, preserving intact habitat patches in USSE and urban developments, and protecting habitat that provide diverse microclimates that could serve as refugia are vital for conserving bee diversity and the services they provide within one of their most diverse ecosystems.

## Author Contributions

**Mark A. Buckner:** conceptualization (lead), data curation (lead), formal analysis (lead), funding acquisition (equal), methodology (lead), software (lead), visualization (lead), writing – original draft (lead), writing – review and editing (lead). **Steven T. Hoge:** data curation (supporting), writing – review and editing (supporting). **Bryan N. Danforth:** conceptualization (supporting), funding acquisition (equal), supervision (lead), writing – review and editing (supporting).

## Conflicts of Interest

The authors declare no conflicts of interest.

## Supporting information


Appendix S1

## Data Availability

The data and scripts used in this study are archived on Figshare: https://doi.org/10.6084/m9.figshare.26081812.

## References

[ece370638-bib-0001] Abatzoglou, J. T. , and C. A. Kolden . 2011. “Climate Change in Western US Deserts: Potential for Increased Wildfire and Invasive Annual Grasses.” Rangeland Ecology & Management 64, no. 5: 471–478. 10.2111/REM-D-09-00151.1.

[ece370638-bib-0002] Amatulli, G. , D. McInerney , T. Sethi , P. Strobl , and S. Domisch . 2020. “Geomorpho90m, Empirical Evaluation and Accuracy Assessment of Global High‐Resolution Geomorphometric Layers.” Scientific Data 7, no. 1: 162. 10.1038/s41597-020-0479-6. 32467582 PMC7256046

[ece370638-bib-0005] Baddeley, A. , E. Rubak , and R. Turner . 2015. Spatial Point Patterns: Methodology and Applications With R. Champan & Hall/CRC Interdisciplinary Statistics Series. Boca Raton, London, New York: CRC Press, Taylor & Francis Group.

[ece370638-bib-0006] Bakx, T. R. M. , M. Green , C. Akselsson , Å. Lindström , Ø. H. Opedal , and H. G. Smith . 2023. “Areas of High Conservation Value Support Specialist Forest Birds.” Ecosphere 14, no. 6: e4559. 10.1002/ecs2.4559.

[ece370638-bib-0007] Balvanera, P. , A. Pfaff , A. Viña , et al. 2019. “Chapter 2.1 Status and Trends—Drivers of Change.” 10.5281/zenodo.5517423.

[ece370638-bib-0008] Barbosa, A. M. , R. Real , A.‐R. Muñoz , and J. A. Brown . 2013. “New Measures for Assessing Model Equilibrium and Prediction Mismatch in Species Distribution Models.” Diversity and Distributions 19, no. 10: 1333–1338. 10.1111/ddi.12100.

[ece370638-bib-0009] Barrett, M. , N. Tigreros , G. Davidowitz , and S. O'Donnell . 2022. “Adaptive Variation in Sex and Male Size Morph Critical Thermal Maxima in Centris Pallida Desert Bees.” 10.2139/ssrn.4085466.

[ece370638-bib-0010] Bartomeus, I. , J. S. Ascher , J. Gibbs , et al. 2013. “Historical Changes in Northeastern US Bee Pollinators Related to Shared Ecological Traits.” Proceedings of the National Academy of Sciences of the United States of America 110, no. 12: 4656–4660. 10.5061/dryad.0nj49. 23487768 PMC3606985

[ece370638-bib-0011] Beier, P. , M. L. Hunter , and M. Anderson . 2015. “Special Section: Conserving Nature's Stage.” Conservation Biology 29, no. 3: 613–617. 10.1111/cobi.12511. 26161444

[ece370638-bib-0012] Bennett, J. M. , J. Sunday , P. Calosi , et al. 2021. “The Evolution of Critical Thermal Limits of Life on Earth.” Nature Communications 12, no. 1: 1198. 10.1038/s41467-021-21263-8. PMC7895938 33608528

[ece370638-bib-0013] Biesmeijer, J. C. , S. P. M. Roberts , M. Reemer , et al. 2006. “Parallel Declines in Pollinators and Insect‐Pollinated Plants in Britain and The Netherlands.” Science 313, no. 5785: 351–354. 10.1126/science.1127863. 16857940

[ece370638-bib-0014] BLM . 2013. “January 2013 Record of Decision and Approved Resource Management Plan Amendments.” US Bureau of Land Management, Washington, DC, USA. https://eplanning.blm.gov/public_projects/nepa/79922/107093/131007/RDEP‐ROD‐ARMP.pdf.

[ece370638-bib-0015] BLM . 2016. “BLM LUPA Record of Decision for the Desert Renewable Energy Conservation Plan and CDCA Amendment.” US Bureau of Land Management, California Office, USA. https://eplanning.blm.gov/public_projects/lup/66459/133460/163124/DRECP_BLM_LUPA_ROD.pdf.

[ece370638-bib-0016] BLM & DOE . 2012. “Final Programmatic Environmental Impact Statement (PEIS) for Solar Energy Development in Six Southwestern States.” US Bureau of Land Management, Washington, DC, USA. https://www.energy.gov/nepa/articles/eis‐0403‐final‐programmatic‐environmental‐impact‐statement.

[ece370638-bib-0017] Bogusch, P. , E. Bláhová , and J. Horák . 2020. “Pollen Specialists Are More Endangered Than Non‐Specialised Bees Even Though They Collect Pollen on Flowers of Non‐Endangered Plants.” Arthropod‐Plant Interactions 14, no. 6: 759–769. 10.1007/s11829-020-09789-y.

[ece370638-bib-0018] Borgelt, J. , M. Dorber , M. A. Høiberg , and F. Verones . 2022. “More Than Half of Data Deficient Species Predicted to Be Threatened by Extinction.” Communications Biology 5, no. 1: 1–9. 10.1038/s42003-022-03638-9. 35927327 PMC9352662

[ece370638-bib-0019] Burdine, J. D. , and K. E. McCluney . 2019. “Differential Sensitivity of Bees to Urbanization‐Driven Changes in Body Temperature and Water Content.” Scientific Reports 9, no. 1: 1643. 10.1038/s41598-018-38338-0. 30733542 PMC6367438

[ece370638-bib-0020] Burkle, L. A. , J. C. Marlin , and T. M. Knight . 2013. “Plant‐Pollinator Interactions Over 120 Years: Loss of Species, Co‐Occurrence, and Function.” Science 339, no. 6127: 1611–1615. 10.1126/science.1232728. 23449999

[ece370638-bib-0021] Cane, J. H. , R. L. Minckley , L. J. Kervin , T. H. Roulston , and N. M. Williams . 2006. “Complex Responses Within A Desert Bee Guild (Hymenoptera: Apiformes) to Urban Habitat Fragmentation.” Ecological Applications 16, no. 2: 632–644. 10.1890/1051-0761(2006)016[0632:CRWADB]2.0.CO;2. 16711050

[ece370638-bib-0022] Capellán‐Pérez, I. , C. de Castro , and I. Arto . 2017. “Assessing Vulnerabilities and Limits in the Transition to Renewable Energies: Land Requirements Under 100% Solar Energy Scenarios.” Renewable and Sustainable Energy Reviews 77: 760–782. 10.1016/j.rser.2017.03.137.

[ece370638-bib-0023] Carril, O. M. , T. Griswold , J. Haefner , and J. S. Wilson . 2018. “Wild Bees of Grand Staircase‐Escalante National Monument: Richness, Abundance, and Spatio‐Temporal Beta‐Diversity.” PeerJ 2018, no. 11: e5867. 10.7717/peerj.5867. PMC6230437 30425889

[ece370638-bib-0024] Ceballos, G. , P. R. Ehrlich , A. D. Barnosky , A. García , R. M. Pringle , and T. M. Palmer . 2015. “Accelerated Modern Human–Induced Species Losses: Entering the Sixth Mass Extinction.” Science Advances 1, no. 5: e1400253. 10.1126/sciadv.1400253. 26601195 PMC4640606

[ece370638-bib-0025] Chappell, M. A. 1982. “Temperature Regulation of Carpenter Bees (*Xylocopa californica*) Foraging in the Colorado Desert of Southern California.” Physiological Zoology 55, no. 3: 267–280.

[ece370638-bib-0026] Chen, G. , X. Li , and X. Liu . 2022. “Global Land Projection Based on Plant Functional Types With a 1‐Km Resolution Under Socio‐Climatic Scenarios.” Scientific Data 9, no. 1: 125. 10.1038/s41597-022-01208-6. 35354830 PMC8967933

[ece370638-bib-0027] Convention on Biological Diversity . 2022. Kunming‐Montreal Global Biodiversity Framework. Montreal, Canada: Convention on Biological Diversity.

[ece370638-bib-0028] Danforth, B. N. 1999. “Emergence Dynamics and Bet Hedging in a Desert Bee, *Perdita portalis* .” Proceedings of the Royal Society of London. Series B: Biological Sciences 266, no. 1432: 1985–1994. 10.1098/rspb.1999.0876.

[ece370638-bib-0029] Deneu, B. , M. Servajean , P. Bonnet , C. Botella , F. Munoz , and A. Joly . 2021. “Convolutional Neural Networks Improve Species Distribution Modelling by Capturing the Spatial Structure of the Environment.” PLoS Computational Biology 17, no. 4: e1008856. 10.1371/journal.pcbi.1008856. 33872302 PMC8084334

[ece370638-bib-0030] Dew, R. M. , D. P. Silva , and S. M. Rehan . 2019. “Range Expansion of an Already Widespread Bee Under Climate Change.” Global Ecology and Conservation 17: e00584. 10.1016/j.gecco.2019.e00584.

[ece370638-bib-0031] Dicks, L. V. , T. D. Breeze , H. T. Ngo , et al. 2021. “A Global‐Scale Expert Assessment of Drivers and Risks Associated With Pollinator Decline.” Nature Ecology & Evolution 5, no. 10: 1453–1461. 10.1038/s41559-021-01534-9. 34400826

[ece370638-bib-0032] Dinerstein, E. , C. Vynne , E. Sala , et al. 2019. “A Global Deal for Nature: Guiding Principles, Milestones, and Targets.” Science Advances 5, no. 4: eaaw2869. 10.1126/sciadv.aaw2869. 31016243 PMC6474764

[ece370638-bib-0033] Donaldson, M. R. , N. J. Burnett , D. C. Braun , et al. 2017. “Taxonomic Bias and International Biodiversity Conservation Research.” FACETS 1: 105–113. 10.1139/facets-2016-0011.

[ece370638-bib-0034] Douglas, M. R. , D. B. Sponsler , E. V. Lonsdorf , and C. M. Grozinger . 2020. “County‐Level Analysis Reveals a Rapidly Shifting Landscape of Insecticide Hazard to Honey Bees (*Apis mellifera*) on US Farmland.” Scientific Reports 10, no. 1: 797. 10.1038/s41598-019-57225-w. 31964921 PMC6972851

[ece370638-bib-0035] Durant, S. M. , N. Pettorelli , S. Bashir , et al. 2012. “Forgotten Biodiversity in Desert Ecosystems.” Science 336, no. 6087: 1379–1380. 10.1126/science.336.6087.1379. 22700901

[ece370638-bib-0036] Elith, J. , M. Kearney , and S. Phillips . 2010. “The Art of Modelling Range‐Shifting Species.” Methods in Ecology and Evolution 1, no. 4: 330–342. 10.1111/j.2041-210x.2010.00036.x.

[ece370638-bib-0037] ESA . 2017. “Land Cover CCI Product User Guide Version 2. Technical Report.” maps.elie.ucl.ac.be/CCI/viewer/download/ESACCI‐LC‐Ph2‐PUGv2_2.0.pdf.

[ece370638-bib-0038] European Commission & Directorate‐General for Environment . 2021. EU Biodiversity Strategy for 2030: Bringing Nature Back Into Our Lives. Luxembourg: Publications Office of the European Union. 10.2779/677548.

[ece370638-bib-0004] Executive Order 14008 . 2021. https://www.govinfo.gov/content/pkg/DCPD‐202100095/pdf/DCPD‐202100095.pdf.

[ece370638-bib-0039] Feng, X. , C. Lin , H. Qiao , and L. Ji . 2015. “Assessment of Climatically Suitable Area for *Syrmaticus reevesii* Under Climate Change.” Endangered Species Research 28, no. 1: 19–31. 10.3354/esr00668.

[ece370638-bib-0040] Gábor, L. , W. Jetz , M. Lu , et al. 2022. “Positional Errors in Species Distribution Modelling Are Not Overcome by the Coarser Grains of Analysis.” Methods in Ecology and Evolution 13, no. 10: 2289–2302. 10.1111/2041-210X.13956.

[ece370638-bib-0041] Garfin, G. M. , P. Gonzalez , D. Breshears , et al. 2018. “Chapter 25: Southwest. Impacts, Risks, and Adaptation in the United States: The Fourth National Climate Assessment, Volume II.” 10.7930/NCA4.2018.CH25.

[ece370638-bib-0042] Gasparatos, A. , C. N. H. Doll , M. Esteban , A. Ahmed , and T. A. Olang . 2017. “Renewable Energy and Biodiversity: Implications for Transitioning to a Green Economy.” Renewable and Sustainable Energy Reviews 70: 161–184. 10.1016/j.rser.2016.08.030.

[ece370638-bib-0043] GBIF.org . 2023. “GBIF Occurrence Download.” 10.15468/dl.xb2hpu.

[ece370638-bib-0044] Gelman, A. , and D. B. Rubin . 1992. “Inference From Iterative Simulation Using Multiple Sequences.” Statistical Science 7, no. 4: 457–472. 10.1214/ss/1177011136.

[ece370638-bib-0045] Golicher, D. , A. Ford , L. Cayuela , and A. Newton . 2012. “Pseudo‐Absences, Pseudo‐Models and Pseudo‐Niches: Pitfalls of Model Selection Based on the Area Under the Curve.” International Journal of Geographical Information Science 26, no. 11: 2049–2063. 10.1080/13658816.2012.719626.

[ece370638-bib-0046] Grodsky, S. M. , J. W. Campbell , and R. R. Hernandez . 2021. “Solar Energy Development Impacts Flower‐Visiting Beetles and Flies in the Mojave Desert.” Biological Conservation 263: 109336. 10.1016/j.biocon.2021.109336.

[ece370638-bib-0047] Grodsky, S. M. , and R. R. Hernandez . 2020. “Reduced Ecosystem Services of Desert Plants From Ground‐Mounted Solar Energy Development.” Nature Sustainability 3, no. 12: 1036–1043. 10.1038/s41893-020-0574-x.

[ece370638-bib-0048] Guerin, G. R. , and A. J. Lowe . 2015. “‘Sum of Inverse Range‐Sizes’ (SIR), a Biodiversity Metric With Many Names and Interpretations.” Biodiversity and Conservation 24, no. 11: 2877–2882. 10.1007/s10531-015-0977-6.

[ece370638-bib-0049] Hamblin, A. L. , E. Youngsteadt , M. M. López‐Uribe , and S. D. Frank . 2017. “Physiological Thermal Limits Predict Differential Responses of Bees to Urban Heat‐Island Effects.” Biology Letters 13, no. 6: 20170125. 10.1098/rsbl.2017.0125. 28637837 PMC5493736

[ece370638-bib-0050] Hantson, S. , T. E. Huxman , S. Kimball , J. T. Randerson , and M. L. Goulden . 2021. “Warming as a Driver of Vegetation Loss in the Sonoran Desert of California.” Journal of Geophysical Research: Biogeosciences 126, no. 6: e2020JG005942. 10.1029/2020JG005942.

[ece370638-bib-0051] Hengl, T. 2018. “Sand Content in % (kg/kg) at 6 Standard Depths (0, 10, 30, 60, 100 and 200 cm) at 250 m Resolution.” 10.5281/zenodo.2525662.

[ece370638-bib-0052] Hirzel, A. H. , G. Le Lay , V. Helfer , C. Randin , and A. Guisan . 2006. “Evaluating the Ability of Habitat Suitability Models to Predict Species Presences.” Ecological Modelling 199, no. 2: 142–152. 10.1016/j.ecolmodel.2006.05.017.

[ece370638-bib-0053] Hostetler, N. E. , and M. E. McIntyre . 2001. “Effects of Urban Land Use on Pollinator (Hymenoptera: Apoidea) Communities in a Desert Metropolis.” Basic and Applied Ecology 2, no. 3: 209–218. 10.1078/1439-1791-00051.

[ece370638-bib-0054] Inman, R. , J. Franklin , T. Esque , and K. Nussear . 2021. “Comparing Sample Bias Correction Methods for Species Distribution Modeling Using Virtual Species.” Ecosphere 12, no. 3: e03422. 10.1002/ecs2.3422.

[ece370638-bib-0055] IPCC . 2022. Climate Change and Land: IPCC Special Report on Climate Change, Desertification, Land Degradation, Sustainable Land Management, Food Security, and Greenhouse Gas Fluxes in Terrestrial Ecosystems. Cambridge, UK: Cambridge University Press. 10.1017/9781009157988.

[ece370638-bib-0056] IPCC . 2023a. Climate Change 2022 – Impacts, Adaptation and Vulnerability: Working Group II Contribution to the Sixth Assessment Report of the Intergovernmental Panel on Climate Change. Cambridge, UK: Cambridge University Press. 10.1017/9781009325844.

[ece370638-bib-0057] IPCC . 2023b. Climate Change 2023 – Synthesis Report. A Report of the Intergovernmental Panel on Climate Change. Contribution of Working Groups I, II and III to the Sixth Assessment Report of the Intergovernmental Panel on Climate Change. Geneva, Switzerland: IPCC.

[ece370638-bib-0058] Iturbide, M. , J. Bedia , S. Herrera , O. del Hierro , M. Pinto , and J. M. Gutiérrez . 2015. “A Framework for Species Distribution Modelling With Improved Pseudo‐Absence Generation.” Ecological Modelling 312: 166–174. 10.1016/j.ecolmodel.2015.05.018.

[ece370638-bib-0059] Jeal, C. , V. Perold , C. L. Seymour , S. Ralston‐Paton , and P. G. Ryan . 2019. “Utility‐Scale Solar Energy Facilities—Effects on Invertebrates in an Arid Environment.” Journal of Arid Environments 168: 1–8. 10.1016/j.jaridenv.2019.05.008.

[ece370638-bib-0060] Jenkins, C. N. , K. S. Van Houtan , S. L. Pimm , and J. O. Sexton . 2015. “US Protected Lands Mismatch Biodiversity Priorities.” National Academy of Sciences of the United States of America 112, no. 16: 5081–5086. 10.1073/pnas.1418034112. PMC4413281 25847995

[ece370638-bib-0061] Johnson, M. G. , K. Alvarez , and J. F. Harrison . 2023. “Water Loss, Not Overheating, Limits the Activity Period of an Endothermic Sonoran Desert Bee.” Functional Ecology 37: 2855–2867. 10.1111/1365-2435.14438.

[ece370638-bib-0062] Johnson, M. G. , J. R. Glass , M. E. Dillon , and J. F. Harrison . 2023. “How Will Climatic Warming Affect Insect Pollinators?” In Advances in Insect Physiology, edited by R. Jurenka , 1–115. Academic Press. 10.1016/bs.aiip.2023.01.001.

[ece370638-bib-0063] Kendall, L. K. , J. M. Mola , Z. M. Portman , D. P. Cariveau , H. G. Smith , and I. Bartomeus . 2022. “The Potential and Realized Foraging Movements of Bees Are Differentially Determined by Body Size and Sociality.” Ecology 103, no. 11: e3809. 10.1002/ecy.3809. 35792515 PMC9786665

[ece370638-bib-0064] Konowalik, K. , and A. Nosol . 2021. “Evaluation Metrics and Validation of Presence‐Only Species Distribution Models Based on Distributional Maps With Varying Coverage.” Scientific Reports 11, no. 1: 1482. 10.1038/s41598-020-80062-1. 33452285 PMC7811024

[ece370638-bib-0065] Kujala, H. , A. Whitehead , and B. Wintle . 2015. “Identifying Conservation Priorities and Assessing Impacts and Trade‐Offs of Potential Future Development in the Lower Hunter Valley in New South Wales.” 10.13140/RG.2.1.1417.4884/1.

[ece370638-bib-0066] Llorente‐Culebras, S. , R. J. Ladle , and A. M. C. Santos . 2023. “Publication Trends in Global Biodiversity Research on Protected Areas.” Biological Conservation 281: 109988. 10.1016/j.biocon.2023.109988.

[ece370638-bib-0067] Mahony, C. R. , T. Wang , A. Hamann , and A. J. Cannon . 2022. “A Global Climate Model Ensemble for Downscaled Monthly Climate Normals Over North America.” International Journal of Climatology 42, no. 11: 5871–5891. 10.1002/joc.7566.

[ece370638-bib-0068] Michener, C. D. 1979. “Biogeography of the Bees.” Annals of the Missouri Botanical Garden 66, no. 3: 277–347.

[ece370638-bib-0069] Minckley, R. L. , J. H. Cane , and L. Kervin . 2000. “Origins and Ecological Consequences of Pollen Specialization Among Desert Bees.” Proceedings of the Royal Society of London. Series B: Biological Sciences 267, no. 1440: 265–271. 10.1098/rspb.2000.0996. PMC1690526 10714881

[ece370638-bib-0070] Minckley, R. L. , and W. R. Radke . 2021. “Extreme Species Density of Bees (Apiformes, Hymenoptera) in the Warm Deserts of North America.” Journal of Hymenoptera Research 82: 317–345. 10.3897/JHR.82.60895.

[ece370638-bib-0071] Minckley, R. L. , T. H. Roulston , and N. M. Williams . 2013. “Resource Assurance Predicts Specialist and Generalist Bee Activity in Drought.” Proceedings of the Royal Society B: Biological Sciences 280, no. 1759: 20122703. 10.1098/rspb.2012.2703. PMC3619498 23536593

[ece370638-bib-0072] Moloney, K. A. , E. L. Mudrak , A. Fuentes‐Ramirez , H. Parag , M. Schat , and C. Holzapfel . 2019. “Increased Fire Risk in Mojave and Sonoran Shrublands Due to Exotic Species and Extreme Rainfall Events.” Ecosphere 10, no. 2: e02592. 10.1002/ecs2.2592.

[ece370638-bib-0073] Munson, S. M. , R. H. Webb , J. Belnap , J. Andrew Hubbard , D. E. Swann , and S. Rutman . 2012. “Forecasting Climate Change Impacts to Plant Community Composition in the Sonoran Desert Region.” Global Change Biology 18, no. 3: 1083–1095. 10.1111/j.1365-2486.2011.02598.x.

[ece370638-bib-0003] National Goal for Renewable Energy Production on Federal Land , 43 U.S.C § 3004. 2020. https://www.govinfo.gov/content/pkg/USCODE‐2023‐title43/pdf/USCODE‐2023‐title43‐chap48‐sec3004.pdf.

[ece370638-bib-0074] Norberg, A. , N. Abrego , F. G. Blanchet , et al. 2019. “A Comprehensive Evaluation of Predictive Performance of 33 Species Distribution Models at Species and Community Levels.” Ecological Monographs 89, no. 3: e01370. 10.1002/ecm.1370.

[ece370638-bib-0075] Omernik, J. M. 1987. “Ecoregions of the Conterminous United States.” Annals of the Association of American Geographers 77, no. 1: 118–125. 10.1111/j.1467-8306.1987.tb00149.x.

[ece370638-bib-0076] Orr, M. C. , A. C. Hughes , D. Chesters , J. Pickering , C. D. Zhu , and J. S. Ascher . 2021. “Global Patterns and Drivers of Bee Distribution.” Current Biology 31, no. 3: 451–458.e4. 10.1016/j.cub.2020.10.053. 33217320

[ece370638-bib-0077] Ovaskainen, O. , and N. Abrego . 2020. Joint Species Distribution Modelling: With Applications in R. Ecology, Biodiversity and Conservation. Cambridge, UK: Cambridge University Press. 10.1017/9781108591720.

[ece370638-bib-0078] Phillips, S. J. , M. Dudík , J. Elith , et al. 2009. “Sample Selection Bias and Presence‐Only Distribution Models: Implications for Background and Pseudo‐Absence Data.” Ecological Applications 19, no. 1: 181–197. 10.1890/07-2153.1. 19323182

[ece370638-bib-0079] Portman, Z. M. , V. J. Tepedino , and A. D. Tripodi . 2019. “Persistence of an Imperiled Specialist Bee and Its Rare Host Plant in a Protected Area.” Insect Conservation and Diversity 12, no. 3: 183–192. 10.1111/icad.12334.

[ece370638-bib-0080] Powney, G. D. , C. Carvell , M. Edwards , et al. 2019. “Widespread Losses of Pollinating Insects in Britain.” Nature Communications 10, no. 1: 1018. 10.1038/s41467-019-08974-9. PMC6435717 30914632

[ece370638-bib-0081] R Core Team . 2022. R: A Language and Environment for Statistical Computing. Vienna, Austria: R Foundation for Statistical Computing. https://www.R‐project.org/.

[ece370638-bib-0082] Raes, N. , and H. ter Steege . 2007. “A Null‐Model for Significance Testing of Presence‐Only Species Distribution Models.” Ecography 30, no. 5: 727–736. 10.1111/j.2007.0906-7590.05041.x.

[ece370638-bib-0083] Riahi, K. , D. P. van Vuuren , E. Kriegler , et al. 2017. “The Shared Socioeconomic Pathways and Their Energy, Land Use, and Greenhouse Gas Emissions Implications: An Overview.” Global Environmental Change 42: 153–168. 10.1016/j.gloenvcha.2016.05.009.

[ece370638-bib-0084] Roberts, D. R. , V. Bahn , S. Ciuti , et al. 2017. “Cross‐Validation Strategies for Data With Temporal, Spatial, Hierarchical, or Phylogenetic Structure.” Ecography 40, no. 8: 913–929. 10.1111/ecog.02881.

[ece370638-bib-0085] Sage, R. F. 2020. “Global Change Biology: A Primer.” Global Change Biology 26, no. 1: 3–30. 10.1111/gcb.14893. 31663217

[ece370638-bib-0086] SCAN . 2022. “Symbiota Collections of Arthropods Network.” https://scan‐bugs.org/portal/index.php.

[ece370638-bib-0087] Senay, S. D. , S. P. Worner , and T. Ikeda . 2013. “Novel Three‐Step Pseudo‐Absence Selection Technique for Improved Species Distribution Modelling.” PLoS One 8, no. 8: e71218. 10.1371/journal.pone.0071218. 23967167 PMC3742778

[ece370638-bib-0088] Shrestha, M. K. , A. M. York , C. G. Boone , and S. Zhang . 2012. “Land Fragmentation Due to Rapid Urbanization in the Phoenix Metropolitan Area: Analyzing the Spatiotemporal Patterns and Drivers.” Applied Geography 32, no. 2: 522–531. 10.1016/j.apgeog.2011.04.004.

[ece370638-bib-0089] Sillero, N. , and A. M. Barbosa . 2021. “Common Mistakes in Ecological Niche Models.” International Journal of Geographical Information Science 35, no. 2: 213–226. 10.1080/13658816.2020.1798968.

[ece370638-bib-0090] Silva, D. P. , R. M. Dew , B. Vilela , M. I. Stevens , and M. P. Schwarz . 2018. “No Deaths in the Desert: Predicted Responses of an Arid‐Adapted Bee and Its Two Nesting Trees Suggest Resilience in the Face of Warming Climates.” Insect Conservation and Diversity 11, no. 5: 449–463. 10.1111/icad.12318.

[ece370638-bib-0091] Stark, J. R. , and J. D. Fridley . 2022. “Microclimate‐Based Species Distribution Models in Complex Forested Terrain Indicate Widespread Cryptic Refugia Under Climate Change.” Global Ecology and Biogeography 31, no. 3: 562–575. 10.1111/geb.13447.

[ece370638-bib-0092] Tikhonov, G. , L. Duan , N. Abrego , et al. 2020. “Computationally Efficient Joint Species Distribution Modeling of Big Spatial Data.” Ecology 101, no. 2: e02929. 10.1002/ecy.2929. 31725922 PMC7027487

[ece370638-bib-0093] Tikhonov, G. , O. Ovaskainen , J. Oksanen , M. de Jonge , O. Opedal , and T. Dallas . 2023. “Hmsc: Hierarchical Model of Species Communities.” https://www.helsinki.fi/en/researchgroups/statistical‐ecology/software/hmsc.

[ece370638-bib-0094] Turley, N. E. , D. J. Biddinger , N. K. Joshi , and M. M. López‐Uribe . 2022. “Six Years of Wild Bee Monitoring Shows Changes in Biodiversity Within and Across Years and Declines in Abundance.” Ecology and Evolution 12, no. 8: e9190. 10.1002/ece3.9190. 35983174 PMC9374588

[ece370638-bib-0095] UNEP‐WCMC . 2023. “Protected Area Profile for United States of America from the World Database on Protected Areas, July 2023.” www.protectedplanet.net.

[ece370638-bib-0096] USGS GAP . 2022. “Protected Areas Database of the United States (PAD‐US).” 10.5066/P9Q9LQ4B.

[ece370638-bib-0097] Valavi, R. , J. Elith , J. J. Lahoz‐Monfort , and G. Guillera‐Arroita . 2019. “blockCV: An R Package for Generating Spatially or Environmentally Separated Folds for k‐Fold Cross‐Validation of Species Distribution Models.” Methods in Ecology and Evolution 10, no. 2: 225–232. 10.1111/2041-210X.13107.

[ece370638-bib-0098] Valavi, R. , J. Elith , J. J. Lahoz‐Monfort , and G. Guillera‐Arroita . 2021. “Modelling Species Presence‐Only Data With Random Forests.” Ecography 44, no. 12: 1731–1742. 10.1111/ecog.05615.

[ece370638-bib-0099] Venter, O. , A. Magrach , N. Outram , et al. 2018. “Bias in Protected‐Area Location and Its Effects on Long‐Term Aspirations of Biodiversity Conventions.” Conservation Biology 32, no. 1: 127–134. 10.1111/cobi.12970. 28639356

[ece370638-bib-0100] Wang, T. , A. Hamann , D. Spittlehouse , and C. Carroll . 2016. “Locally Downscaled and Spatially Customizable Climate Data for Historical and Future Periods for North America.” PLoS One 11, no. 6: e0156720. 10.1371/journal.pone.0156720. 27275583 PMC4898765

[ece370638-bib-0101] Williams, A. P. , E. R. Cook , J. E. Smerdon , et al. 2020. “Large Contribution From Anthropogenic Warming to an Emerging North American Megadrought.” Science 368, no. 6488: 314–318. 10.1126/science.aaz9600. 32299953

[ece370638-bib-0102] Wood, T. J. , J. M. Holland , and D. Goulson . 2016. “Diet Characterisation of Solitary Bees on Farmland: Dietary Specialisation Predicts Rarity.” Biodiversity and Conservation 25, no. 13: 2655–2671. 10.1007/s10531-016-1191-x. 32355425 PMC7175682

[ece370638-bib-0103] Woodard, S. H. , S. Federman , R. R. James , et al. 2020. “Towards a U.S. National Program for Monitoring Native Bees.” Biological Conservation 252: 108821. 10.1016/j.biocon.2020.108821.

[ece370638-bib-0104] Wright, M. N. , and A. Ziegler . 2017. “Ranger: A Fast Implementation of Random Forests for High Dimensional Data in C++ and R.” Journal of Statistical Software 77: 1–17. 10.18637/jss.v077.i01.

[ece370638-bib-0105] Wu, J. , G. D. Jenerette , A. Buyantuyev , and C. L. Redman . 2011. “Quantifying Spatiotemporal Patterns of Urbanization: The Case of the Two Fastest Growing Metropolitan Regions in the United States.” Ecological Complexity 8, no. 1: 1–8. 10.1016/j.ecocom.2010.03.002.

[ece370638-bib-0106] Zizka, A. , D. Silvestro , T. Andermann , et al. 2019. “CoordinateCleaner: Standardized Cleaning of Occurrence Records From Biological Collection Databases.” Methods in Ecology and Evolution 10, no. 5: 744–751. 10.1111/2041-210X.13152.

