# Supplementary material for: Forecasting the Effects of Global Change on a Bee Biodiversity Hotspot
Source: Ecol Evol. 2024 Nov 29;14(12):e70638. doi: 10.1002/ece3.70638 (PMC11606700; doi:10.1002/ece3.70638)
Supplement: Supplementary file 1 — Appendix S1 [file ECE3-14-e70638-s001.docx]

**Supplement: Forecasting the Effects of Global Change on a Bee Biodiversity Hotspot**

Mark A. Buckner*^a^, Steven T. Hoge^a^, Bryan N. Danforth^a^

^a^ Cornell University, Department of Entomology, 2126 Comstock Hall, Ithaca, New York, USA 14853

*Corresponding Author: [mab677@cornell.edu](mailto:mab677@cornell.edu)

**Table S1:** Trait data for all modeled bee species

| **Species** | **Subgenus** | **Sociality** | **Parasite** | **Nest** | **Specialist** | **Oil Collection** | **Over-wintering** | **Origin** | **Diurnality** | **References** |
| --- | --- | --- | --- | --- | --- | --- | --- | --- | --- | --- |
| *Agapostemon angelicus* | Agapostemon | Solitary | No | ground | No | No | Adult | Native | Diurnal | [15](#fifteen), [16](#sixteen), [19](#nineteen), [24](#twenty4) |
| *Agapostemon melliventris* | Agapostemon | Solitary | No | ground | No | No | Adult | Native | Diurnal | [15](#fifteen), [16](#sixteen), [19](#nineteen), [24](#twenty4) |
| *Ancylandrena larreae* | - | Solitary | No | ground | Yes | No | Prepupa | Native | Diurnal | [15](#fifteen), [16](#sixteen), [23](#twenty3), [24](#twenty4), [45](#forty5), [46](#forty6) |
| *Ancylandrena timberlakei* | - | Solitary | No | ground | No | No | Prepupa | Native | Diurnal | [15](#fifteen), [16](#sixteen), [24](#twenty4), [46](#forty6) |
| *Andrena cerasifolii* | - | Solitary | No | ground | No | No | Adult | Native | Diurnal | [15](#fifteen), [16](#sixteen), [24](#twenty4) |
| *Andrena fracta* | Plastandrena | Solitary | No | ground | No | No | Adult | Native | Diurnal | [15](#fifteen), [16](#sixteen), [24](#twenty4), [34](#thirty4) |
| *Andrena palpalis* | Belandrena | Solitary | No | ground | No | No | Adult | Native | Diurnal | [15](#fifteen), [16](#sixteen), [24](#twenty4), [34](#thirty4) |
| *Andrena prunorum* | Plastandrena | Solitary | No | ground | No | No | Adult | Native | Diurnal | [15](#fifteen), [16](#sixteen), [24](#twenty4), [32](#thirty2) |
| *Andrena sphaeralceae* | - | Solitary | No | ground | Yes | No | Adult | Native | Diurnal | [15](#fifteen), [16](#sixteen), [24](#twenty4), [25](#twenty5) |
| *Anthidiellum ehrhorni* | Loyolanthidium | Solitary | No | above | No | No | Prepupa | Native | Diurnal | [15](#fifteen), [16](#sixteen), [24](#twenty4) |
| *Anthidiellum notatum* | Loyolanthidium | Solitary | No | above | No | No | Prepupa | Native | Diurnal | [15](#fifteen), [16](#sixteen), [24](#twenty4) |
| *Anthidiellum robertsoni* | Loyolanthidium | Solitary | No | above | No | No | Prepupa | Native | Diurnal | [15](#fifteen), [16](#sixteen), [24](#twenty4) |
| *Anthidium cockerelli* | Anthidium | Solitary | No | ground | No | No | Prepupa | Native | Diurnal | [15](#fifteen), [16](#sixteen), [24](#twenty4) |
| *Anthidium emarginatum* | Anthidium | Solitary | No | ground | No | No | Prepupa | Native | Diurnal | [15](#fifteen), [16](#sixteen), [24](#twenty4) |
| *Anthidium jocosum* | Anthidium | Solitary | No | ground | No | No | Prepupa | Native | Diurnal | [15](#fifteen), [16](#sixteen), [24](#twenty4) |
| *Anthidium maculosum* | Anthidium | Solitary | No | above | No | No | Prepupa | Native | Diurnal | [15](#fifteen), [16](#sixteen), [24](#twenty4) |
| *Anthidium palmarum* | Anthidium | Solitary | No | ground | No | No | Prepupa | Native | Diurnal | [15](#fifteen), [16](#sixteen), [24](#twenty4) |
| *Anthidium paroselae* | Anthidium | Solitary | No | ground | No | No | Prepupa | Native | Diurnal | [15](#fifteen), [16](#sixteen), [24](#twenty4) |
| *Anthophora californica* | Anthophoroides | Solitary | No | ground | No | No | Prepupa | Native | Diurnal | [15](#fifteen), [16](#sixteen), [24](#twenty4), [63](#sixty3) |
| *Anthophora centriformis* | Paramegilla | Solitary | No | ground | No | No | Prepupa | Native | Diurnal | [15](#fifteen), [16](#sixteen), [24](#twenty4), [63](#sixty3) |
| *Anthophora cockerelli* | Micranthophora | Solitary | No | ground | No | No | Prepupa | Native | Diurnal | [15](#fifteen), [16](#sixteen), [24](#twenty4), [63](#sixty3) |
| *Anthophora coptognatha* | Lophanthophora | Solitary | No | ground | No | No | Prepupa | Native | Diurnal | [15](#fifteen), [16](#sixteen), [24](#twenty4), [63](#sixty3) |
| *Anthophora curta* | Micranthophora | Solitary | No | ground | No | No | Prepupa | Native | Diurnal | [15](#fifteen), [16](#sixteen), [24](#twenty4), [63](#sixty3) |
| *Anthophora hololeuca* | Micranthophora | Solitary | No | ground | No | No | Prepupa | Native | Diurnal | [15](#fifteen), [16](#sixteen), [24](#twenty4), [63](#sixty3) |
| *Anthophora neglecta* | Lophanthophora | Solitary | No | ground | No | No | Prepupa | Native | Diurnal | [15](#fifteen), [16](#sixteen), [24](#twenty4), [63](#sixty3) |
| *Anthophora pachyodonta* | Micranthophora | Solitary | No | ground | No | No | Prepupa | Native | Diurnal | [15](#fifteen), [16](#sixteen), [24](#twenty4), [63](#sixty3) |
| *Anthophora petrophila* | Micranthophora | Solitary | No | ground | No | No | Prepupa | Native | Diurnal | [15](#fifteen), [16](#sixteen), [24](#twenty4), [63](#sixty3) |
| *Anthophora phenax* | Micranthophora | Solitary | No | ground | No | No | Prepupa | Native | Diurnal | [15](#fifteen), [16](#sixteen), [24](#twenty4), [63](#sixty3) |
| *Anthophora urbana* | Mystacanthophora | Solitary | No | ground | No | No | Prepupa | Native | Diurnal | [15](#fifteen), [16](#sixteen), [24](#twenty4), [63](#sixty3) |
| *Anthophora vannigera* | Pyganthophora | Solitary | No | ground | No | No | Prepupa | Native | Diurnal | [15](#fifteen), [16](#sixteen), [24](#twenty4) |
| *Apis mellifera* | Apis | Social | No | above | No | No | Adult | Non-Native | Diurnal | [15](#fifteen), [16](#sixteen), [24](#twenty4) |
| *Ashmeadiella bigeloviae* | Ashmeadiella | Solitary | No | above | No | No | Prepupa | Native | Diurnal | [15](#fifteen), [16](#sixteen), [24](#twenty4), [43](#forty3) |
| *Ashmeadiella breviceps* | Arogochila | Solitary | No | above | No | No | Prepupa | Native | Diurnal | [15](#fifteen), [16](#sixteen), [24](#twenty4), [43](#forty3) |
| *Ashmeadiella bucconis* | Ashmeadiella | Solitary | No | above | Yes | No | Prepupa | Native | Diurnal | [15](#fifteen), [16](#sixteen), [23](#twenty3), [24](#twenty4), [43](#forty3) |
| *Ashmeadiella femorata* | Ashmeadiella | Solitary | No | above | No | No | Prepupa | Native | Diurnal | [15](#fifteen), [16](#sixteen), [24](#twenty4), [43](#forty3) |
| *Ashmeadiella meliloti* | Ashmeadiella | Solitary | No | above | No | No | Prepupa | Native | Diurnal | [15](#fifteen), [16](#sixteen), [24](#twenty4), [43](#forty3) |
| *Ashmeadiella prosopidis* | Ashmeadiella | Solitary | No | above | No | No | Prepupa | Native | Diurnal | [15](#fifteen), [16](#sixteen), [24](#twenty4), [43](#forty3) |
| *Ashmeadiella rhodognatha* | Chilosima | Solitary | No | above | No | No | Prepupa | Native | Diurnal | [15](#fifteen), [16](#sixteen), [24](#twenty4), [43](#forty3) |
| *Augochlorella pomoniella* | - | Social | No | ground | No | No | Adult | Native | Diurnal | [9](#nine),[15](#fifteen), [16](#sixteen), [24](#twenty4) |
| *Bombus californicus* | Thoracobombus | Social | No | ground | No | No | Adult | Native | Diurnal | [15](#fifteen), [16](#sixteen), [24](#twenty4) |
| *Bombus crotchii* | Cullumanobombus | Social | No | ground | No | No | Adult | Native | Diurnal | [15](#fifteen), [16](#sixteen), [24](#twenty4) |
| *Bombus huntii* | Pyrobombus | Social | No | ground | No | No | Adult | Native | Diurnal | [15](#fifteen), [16](#sixteen), [24](#twenty4) |
| *Bombus melanopygus* | Pyrobombus | Social | No | ground | No | No | Adult | Native | Diurnal | [15](#fifteen), [16](#sixteen), [24](#twenty4) |
| *Bombus morrisoni* | Cullumanobombus | Social | No | ground | No | No | Adult | Native | Diurnal | [15](#fifteen), [16](#sixteen), [24](#twenty4) |
| *Bombus pensylvanicus* | Thoracobombus | Social | No | ground | No | No | Adult | Native | Diurnal | [15](#fifteen), [16](#sixteen), [24](#twenty4) |
| *Bombus sonorus* | Thoracobombus | Social | No | ground | No | No | Adult | Native | Diurnal | [15](#fifteen), [16](#sixteen), [24](#twenty4) |
| *Bombus vandykei* | Pyrobombus | Social | No | ground | No | No | Adult | Native | Diurnal | [15](#fifteen), [16](#sixteen), [24](#twenty4) |
| *Bombus vosnesenskii* | Pyrobombus | Social | No | ground | No | No | Adult | Native | Diurnal | [15](#fifteen), [16](#sixteen), [24](#twenty4) |
| *Brachymelecta californica* | - | Solitary | Yes | ground | No | No | Prepupa | Native | Diurnal | [15](#fifteen), [16](#sixteen), [24](#twenty4), [36](#thirty6) |
| *Calliopsis anomoptera* | Perissander | Solitary | No | ground | Yes | No | Prepupa | Native | Diurnal | [15](#fifteen), [16](#sixteen), [24](#twenty4), [59](#fifty9) |
| *Calliopsis puellae* | Nomadopsis | Solitary | No | ground | Yes | No | Prepupa | Native | Diurnal | [15](#fifteen), [16](#sixteen), [24](#twenty4), [59](#fifty9) |
| *Calliopsis rozeni* | Calliopsima | Solitary | No | ground | Yes | No | Prepupa | Native | Diurnal | [15](#fifteen), [16](#sixteen), [24](#twenty4), [59](#fifty9) |
| *Calliopsis subalpina* | Hypomacrotera | Solitary | No | ground | Yes | No | Prepupa | Native | Diurnal | [15](#fifteen), [16](#sixteen), [24](#twenty4), [59](#fifty9) |
| *Centris atripes* | Paracentris | Solitary | No | ground | No | Yes | Prepupa | Native | Diurnal | [15](#fifteen), [16](#sixteen), [20](#twenty), [24](#twenty4) |
| *Centris cockerelli* | Paracentris | Solitary | No | ground | No | Yes | Prepupa | Native | Diurnal | [15](#fifteen), [16](#sixteen), [20](#twenty), [24](#twenty4) |
| *Centris hoffmanseggiae* | Paracentris | Solitary | No | ground | No | No | Prepupa | Native | Diurnal | [15](#fifteen), [16](#sixteen), [20](#twenty), [24](#twenty4) |
| *Centris pallida* | Paracentris | Solitary | No | ground | No | No | Prepupa | Native | Diurnal | [8](#eight), [15](#fifteen), [16](#sixteen), [20](#twenty), [24](#twenty4) |
| *Centris rhodopus* | Paracentris | Solitary | No | ground | No | Yes | Prepupa | Native | Diurnal | [15](#fifteen), [16](#sixteen), [20](#twenty), [24](#twenty4) |
| *Ceratina apacheorum* | Zadontomerus | Solitary | No | above | No | No | Adult | Native | Diurnal | [15](#fifteen), [16](#sixteen), [24](#twenty4) |
| *Ceratina arizonensis* | Ceratinula | Solitary | No | above | No | No | Adult | Native | Diurnal | [15](#fifteen), [16](#sixteen), [24](#twenty4), [41](#forty1) |
| *Chelostoma californicum* | Neochelostoma | Solitary | No | above | Yes | No | Prepupa | Native | Diurnal | [15](#fifteen), [16](#sixteen), [24](#twenty4) |
| *Colletes cercidii* | - | Solitary | No | ground | No | No | Prepupa | Native | Diurnal | [15](#fifteen), [16](#sixteen), [24](#twenty4), [60](#sixty), [61](#sixty1) |
| *Colletes clypeonitens* | - | Solitary | No | ground | Yes | No | Prepupa | Native | Diurnal | [15](#fifteen), [16](#sixteen), [24](#twenty4) |
| *Colletes larreae* | - | Solitary | No | ground | Yes | No | Prepupa | Native | Diurnal | [15](#fifteen), [16](#sixteen), [23](#twenty3), [24](#twenty4) |
| *Colletes louisae* | - | Solitary | No | ground | No | No | Prepupa | Native | Diurnal | [15](#fifteen), [16](#sixteen), [24](#twenty4) |
| *Colletes salicicola* | - | Solitary | No | ground | No | No | Prepupa | Native | Diurnal | [15](#fifteen), [16](#sixteen), [24](#twenty4) |
| *Colletes stepheni* | - | Solitary | No | ground | Yes | No | Prepupa | Native | Nocturnal | [15](#fifteen), [14](#fourteen), [16](#sixteen), [24](#twenty4) |
| *Conanthalictus bakeri* | - | Solitary | No | ground | Yes | No | Prepupa | Native | Diurnal | [15](#fifteen), [16](#sixteen), [24](#twenty4), [52](#fifty2) |
| *Conanthalictus caerulescens* | - | Solitary | No | ground | Yes | No | Prepupa | Native | Diurnal | [15](#fifteen), [16](#sixteen), [24](#twenty4), [52](#fifty2) |
| *Diadasia australis* | Coquillettapis | Solitary | No | ground | Yes | No | Prepupa | Native | Diurnal | [15](#fifteen), [16](#sixteen), [24](#twenty4) |
| *Diadasia bituberculata* | Coquillettapis | Solitary | No | ground | Yes | No | Prepupa | Native | Diurnal | [15](#fifteen), [16](#sixteen), [24](#twenty4) |
| *Diadasia diminuta* | Coquillettapis | Solitary | No | ground | Yes | No | Prepupa | Native | Diurnal | [15](#fifteen), [16](#sixteen), [24](#twenty4) |
| *Diadasia lutzi* | Coquillettapis | Solitary | No | ground | Yes | No | Prepupa | Native | Diurnal | [15](#fifteen), [16](#sixteen), [24](#twenty4) |
| *Diadasia martialis* | Coquillettapis | Solitary | No | ground | Yes | No | Prepupa | Native | Diurnal | [15](#fifteen), [16](#sixteen), [24](#twenty4) |
| *Diadasia rinconis* | Coquillettapis | Solitary | No | ground | Yes | No | Prepupa | Native | Diurnal | [15](#fifteen), [16](#sixteen), [24](#twenty4), [33](#thirty3),[37](#thirty7) |
| *Diadasia tuberculifrons* | Coquillettapis | Solitary | No | ground | Yes | No | Prepupa | Native | Diurnal | [15](#fifteen), [16](#sixteen), [24](#twenty4), [33](#thirty3) |
| *Diadasia vallicola* | Coquillettapis | Solitary | No | ground | Yes | No | Prepupa | Native | Diurnal | [15](#fifteen), [16](#sixteen), [24](#twenty4), [33](#thirty3) |
| *Dianthidium dubium* | Dianthidium | Solitary | No | above | No | No | Prepupa | Native | Diurnal | [15](#fifteen), [16](#sixteen), [24](#twenty4) |
| *Dianthidium pudicum* | Dianthidium | Solitary | No | above | No | No | Prepupa | Native | Diurnal | [15](#fifteen), [16](#sixteen), [24](#twenty4) |
| *Dianthidium ulkei* | Dianthidium | Solitary | No | above | No | No | Prepupa | Native | Diurnal | [15](#fifteen), [16](#sixteen), [21](#twenty1), [24](#twenty4) |
| *Dieunomia nevadensis* | Epinomia | Solitary | No | ground | No | No | Prepupa | Native | Diurnal | [11](#eleven), [15](#fifteen), [16](#sixteen), [24](#twenty4), [67](#sixty7) |
| *Dioxys productus* | - | Solitary | Yes | above | No | No | Prepupa | Native | Diurnal | [15](#fifteen), [16](#sixteen), [24](#twenty4) |
| *Dufourea mulleri* | - | Solitary | No | ground | Yes | No | Prepupa | Native | Diurnal | [15](#fifteen), [16](#sixteen), [24](#twenty4), [66](#sixty6) |
| *Epeolus mesillae* | - | Solitary | Yes | ground | Yes | No | Prepupa | Native | Diurnal | [15](#fifteen), [16](#sixteen), [24](#twenty4), [35](#thirty5) |
| *Ericrocis lata* | - | Solitary | Yes | ground | No | No | Prepupa | Native | Diurnal | [15](#fifteen), [16](#sixteen), [24](#twenty4), [49](#forty9) |
| *Eucera mohavensis* | Synhalonia | Solitary | No | ground | No | No | Prepupa | Native | Diurnal | [15](#fifteen), [16](#sixteen), [17](#seventeen), [24](#twenty4), [62](#sixty2) |
| *Habropoda pallida* | - | Solitary | No | ground | Yes | No | Prepupa | Native | Diurnal | [2](#two), [15](#fifteen), [16](#sixteen), [24](#twenty4) |
| *Habropoda tristissima* | - | Solitary | No | ground | No | No | Prepupa | Native | Diurnal | [2](#two), [15](#fifteen), [16](#sixteen), [24](#twenty4) |
| *Halictus farinosus* | Nealictus | Social | No | ground | No | No | Adult | Native | Diurnal | [15](#fifteen), [16](#sixteen), [24](#twenty4), [30](#thirty) |
| *Halictus ligatus* | Odontalictus | Social | No | ground | No | No | Adult | Native | Diurnal | [15](#fifteen), [16](#sixteen), [24](#twenty4), [30](#thirty) |
| *Halictus tripartitus* | Seladonia | Social | No | ground | No | No | Adult | Native | Diurnal | [15](#fifteen), [16](#sixteen), [24](#twenty4), [30](#thirty), [38](#thirty8) |
| *Hesperapis fuchsi* | Panurgomia | Solitary | No | ground | Yes | No | Prepupa | Native | Diurnal | [15](#fifteen), [16](#sixteen), [24](#twenty4) |
| *Hesperapis larreae* | Amblyapis | Solitary | No | ground | Yes | No | Prepupa | Native | Diurnal | [15](#fifteen), [16](#sixteen), [23](#twenty3), [24](#twenty4), [53](#fifty3) |
| *Hexepeolus rhodogyne* | - | Solitary | Yes | ground | Yes | No | Prepupa | Native | Diurnal | [15](#fifteen), [16](#sixteen), [24](#twenty4), [45](#forty5) |
| *Hoplitis biscutellae* | Alcidamea | Solitary | No | above | Yes | No | Prepupa | Native | Diurnal | [15](#fifteen), [16](#sixteen), [24](#twenty4), [27](#twenty7) |
| *Hoplitis producta* | Alcidamea | Solitary | No | above | No | No | Prepupa | Native | Diurnal | [15](#fifteen), [16](#sixteen), [24](#twenty4), [27](#twenty7) |
| *Hylaeus asininus* | Paraprosopis | Solitary | No | above | No | No | Prepupa | Native | Diurnal | [15](#fifteen), [16](#sixteen), [24](#twenty4), [64](#sixty4) |
| *Hylaeus episcopalis* | Prosopis | Solitary | No | above | No | No | Prepupa | Native | Diurnal | [15](#fifteen), [16](#sixteen), [24](#twenty4) |
| *Hylaeus wootoni* | Paraprosopis | Solitary | No | above | No | No | Prepupa | Native | Diurnal | [15](#fifteen), [16](#sixteen), [24](#twenty4) |
| *Lasioglossum hyalinum* | Dialictus | Social | No | ground | No | No | Adult | Native | Diurnal | [15](#fifteen), [16](#sixteen), [24](#twenty4), [56](#fifty6) |
| *Lasioglossum microlepoides* | Dialictus | Social | No | ground | No | No | Adult | Native | Diurnal | [15](#fifteen), [16](#sixteen), [24](#twenty4), [56](#fifty6) |
| *Lasioglossum perparvum* | Dialictus | Social | No | ground | No | No | Adult | Native | Diurnal | [15](#fifteen), [16](#sixteen), [24](#twenty4), [56](#fifty6) |
| *Lasioglossum pseudotegulare* | Dialictus | Social | No | ground | No | No | Adult | Native | Diurnal | [15](#fifteen), [16](#sixteen), [24](#twenty4), [56](#fifty6) |
| *Lasioglossum sisymbrii* | Lasioglossum | Solitary | No | ground | No | No | Adult | Native | Diurnal | [15](#fifteen), [16](#sixteen), [24](#twenty4), [56](#fifty6) |
| *Lasioglossum stictaspis* | Dialictus | Social | No | ground | No | No | Adult | Native | Diurnal | [15](#fifteen), [16](#sixteen), [24](#twenty4), [56](#fifty6) |
| *Lithurgopsis apicalis* | - | Solitary | No | above | No | No | Prepupa | Native | Diurnal | [15](#fifteen), [16](#sixteen), [24](#twenty4), [47](#forty7) |
| *Lithurgopsis echinocacti* | - | Solitary | No | above | No | No | Prepupa | Native | Diurnal | [15](#fifteen), [16](#sixteen), [24](#twenty4), [47](#forty7) |
| *Macrotera arcuata* | Macroteropsis | Solitary | No | ground | Yes | No | Prepupa | Native | Diurnal | [13](#thirteen), [15](#fifteen), [16](#sixteen), [24](#twenty4) |
| *Macrotera mellea* | Macroterella | Solitary | No | ground | No | No | Prepupa | Native | Diurnal | [13](#thirteen), [15](#fifteen), [16](#sixteen), [24](#twenty4) |
| *Megachile chilopsidis* | Chelostomoides | Solitary | No | above | No | No | Prepupa | Native | Diurnal | [3](#three), [15](#fifteen), [16](#sixteen), [24](#twenty4), [28](#twenty8) |
| *Megachile discorhina* | Chelostomoides | Solitary | No | above | No | No | Prepupa | Native | Diurnal | [15](#fifteen), [16](#sixteen), [24](#twenty4), [28](#twenty8) |
| *Megachile fucata* | Megachiloides | Solitary | No | above | No | No | Prepupa | Native | Diurnal | [15](#fifteen), [16](#sixteen), [24](#twenty4), [28](#twenty8) |
| *Megachile lippiae* | Litomegachile | Solitary | No | above | No | No | Prepupa | Native | Diurnal | [15](#fifteen), [16](#sixteen), [24](#twenty4), [28](#twenty8) |
| *Megachile newberryae* | Sayapis | Solitary | No | above | No | No | Prepupa | Native | Diurnal | [15](#fifteen), [16](#sixteen), [24](#twenty4), [28](#twenty8) |
| *Megachile odontostoma* | Chelostomoides | Solitary | No | above | No | No | Prepupa | Native | Diurnal | [15](#fifteen), [16](#sixteen), [24](#twenty4) , [28](#twenty8) |
| *Megachile policaris* | Sayapis | Solitary | No | above | No | No | Prepupa | Native | Diurnal | [15](#fifteen), [16](#sixteen), [24](#twenty4), [28](#twenty8) |
| *Megachile sidalceae* | Pseudocentron | Solitary | No | above | No | No | Prepupa | Native | Diurnal | [15](#fifteen), [16](#sixteen), [24](#twenty4), [28](#twenty8) |
| *Megandrena enceliae* | Megandrena | Solitary | No | ground | Yes | No | Prepupa | Native | Diurnal | [15](#fifteen), [16](#sixteen), [24](#twenty4) |
| *Melissodes paroselae* | Melissodes | Solitary | No | ground | No | No | Prepupa | Native | Diurnal | [15](#fifteen), [16](#sixteen), [24](#twenty4), [40](#forty) |
| *Melissodes tristis* | Eumelissodes | Solitary | No | ground | No | No | Prepupa | Native | Diurnal | [15](#fifteen), [16](#sixteen), [24](#twenty4), [40](#forty) |
| *Neolarra californica* | - | Solitary | Yes | ground | Yes | No | Prepupa | Native | Diurnal | [15](#fifteen), [16](#sixteen), [23](#twenty3), [24](#twenty4) |
| *Nomia tetrazonata* | Acunomia | Solitary | No | ground | No | No | Prepupa | Native | Diurnal | [15](#fifteen), [16](#sixteen), [24](#twenty4), [67](#sixty7) |
| *Osmia aglaia* | Melanosmia | Solitary | No | above | No | No | Adult | Native | Diurnal | [4](#four), [15](#fifteen), [16](#sixteen), [24](#twenty4), [55](#fifty5) |
| *Osmia ribifloris* | Osmia | Solitary | No | above | No | No | Adult | Native | Diurnal | [[4](#four), 15](#fifteen), [16](#sixteen), [24](#twenty4) |
| *Perdita albonotata* | Procockerellia | Solitary | No | ground | Yes | No | Prepupa | Native | Diurnal | [12](#twelve), [15](#fifteen), [16](#sixteen), [24](#twenty4), [31](#thirty1) |
| *Perdita arenaria* | Heteroperdita | Solitary | No | ground | Yes | No | Prepupa | Native | Diurnal | [15](#fifteen), [16](#sixteen), [24](#twenty4), [31](#thirty1) |
| *Perdita callicerata* | Hexaperdita | Solitary | No | ground | Yes | No | Prepupa | Native | Diurnal | [15](#fifteen), [16](#sixteen), [24](#twenty4), [31](#thirty1) |
| *Perdita coldeniae* | Heteroperdita | Solitary | No | ground | Yes | No | Prepupa | Native | Diurnal | [15](#fifteen), [16](#sixteen), [24](#twenty4), [31](#thirty1) |
| *Perdita covilleae* | Perdita | Solitary | No | ground | Yes | No | Prepupa | Native | Diurnal | [15](#fifteen), [16](#sixteen), [24](#twenty4), [31](#thirty1) |
| *Perdita koebelei* | Perdita | Solitary | No | ground | Yes | No | Prepupa | Native | Diurnal | [15](#fifteen), [16](#sixteen), [24](#twenty4), [31](#thirty1) |
| *Perdita larreae* | Perditella | Solitary | No | ground | Yes | No | Prepupa | Native | Diurnal | [15](#fifteen), [16](#sixteen), [23](#twenty3), [24](#twenty4), [31](#thirty1) |
| *Perdita lateralis* | Perdita | Solitary | No | ground | Yes | No | Prepupa | Native | Diurnal | [15](#fifteen), [16](#sixteen), [24](#twenty4), [31](#thirty1) |
| *Perdita malacothricis* | Pygoperdita | Solitary | No | ground | Yes | No | Prepupa | Native | Diurnal | [15](#fifteen), [16](#sixteen), [24](#twenty4), [31](#thirty1) |
| *Perdita minima* | Perditella | Solitary | No | ground | Yes | No | Prepupa | Native | Diurnal | [15](#fifteen), [16](#sixteen), [24](#twenty4), [31](#thirty1) |
| *Perdita mohavensis* | Pygoperdita | Solitary | No | ground | Yes | No | Prepupa | Native | Diurnal | [15](#fifteen), [16](#sixteen), [24](#twenty4), [31](#thirty1) |
| *Perdita punctosignata* | Perdita | Solitary | No | ground | Yes | No | Prepupa | Native | Diurnal | [15](#fifteen), [16](#sixteen), [24](#twenty4), [31](#thirty1) |
| *Perdita punctulata* | Perdita | Solitary | No | ground | Yes | No | Prepupa | Native | Diurnal | [15](#fifteen), [16](#sixteen), [24](#twenty4), [31](#thirty1) |
| *Pseudomacrotera turgiceps* | - | Solitary | No | ground | Yes | No | Prepupa | Native | Diurnal | [15](#fifteen), [16](#sixteen), [24](#twenty4) |
| *Protodufourea eickworti* | - | Solitary | No | ground | Yes | No | Prepupa | Native | Diurnal | [15](#fifteen), [16](#sixteen), [24](#twenty4), [54](#fifty4) |
| *Protohalonia amoena* | - | Solitary | No | ground | Yes | No | Prepupa | Native | Nocturnal | [14](#fourteen), [15](#fifteen), [16](#sixteen), [24](#twenty4) |
| *Protosmia rubifloris* | Chelostomopsis | Solitary | No | above | No | No | Adult | Native | Diurnal | [15](#fifteen), [16](#sixteen), [22](#twenty2), [24](#twenty4) |
| *Protoxaea gloriosa* | - | Solitary | No | ground | No | No | Prepupa | Native | Diurnal | [15](#fifteen), [16](#sixteen), [24](#twenty4) |
| *Stelis perpulchra* | Dolichostelis | Solitary | Yes | above | No | No | Prepupa | Native | Diurnal | [15](#fifteen), [16](#sixteen), [24](#twenty4) |
| *Epimelissodes duplocincta* | Idiomelissodes | Solitary | No | ground | Yes | No | Prepupa | Native | Diurnal | [15](#fifteen), [16](#sixteen), [24](#twenty4) |
| *Epimelissodes sabinensis* | Epimelissodes | Solitary | No | ground | No | No | Prepupa | Native | Diurnal | [15](#fifteen), [16](#sixteen), [24](#twenty4) |
| *Townsendiella pulchra* | - | Solitary | Yes | ground | Yes | No | Prepupa | Native | Diurnal | [15](#fifteen), [16](#sixteen), [24](#twenty4), [53](#fifty3) |
| *Trachusa larreae* | Heteranthidium | Solitary | No | ground | Yes | No | Prepupa | Native | Diurnal | [15](#fifteen), [16](#sixteen), [23](#twenty3), [24](#twenty4), [50](#fifty) |
| *Triepeolus verbesinae* | - | Solitary | Yes | ground | No | No | Adult | Native | Diurnal | [15](#fifteen), [16](#sixteen), [24](#twenty4) |
| *Xeralictus bicuspidariae* | - | Solitary | No | ground | No | No | Prepupa | Native | Diurnal | [15](#fifteen), [16](#sixteen), [24](#twenty4), [42](#forty2) |
| *Xeralictus timberlakei* | - | Solitary | No | ground | No | No | Prepupa | Native | Diurnal | [15](#fifteen), [16](#sixteen), [24](#twenty4), [42](#forty2) |
| *Xylocopa californica* | Xylocopoides | Solitary | No | above | No | No | Prepupa | Native | Diurnal | [[1](#one), 15](#fifteen), [16](#sixteen), [24](#twenty4) |
| *Xylocopa sonorina* | Neoxylocopa | Solitary | No | above | No | No | Prepupa | Native | Diurnal | [1](#one), [15](#fifteen), [16](#sixteen), [24](#twenty4), [58](#fifty8) |
| *Xylocopa tabaniformis* | Notoxylocopa | Solitary | No | above | No | No | Prepupa | Native | Diurnal | [1](#one), [15](#fifteen), [16](#sixteen), [24](#twenty4) |

**Sociality**, all species exhibiting a degree of social behavior from eusociality to cooperative breeding are marked social. Communally nesting bees are considered solitary. **Parasite** indicates species which parasitize nests of other bee species. **Nest** categorizes species into two broad groups- those that make nests below ground (ground) and those that nest above ground (above) including species nesting in cavities, stems, or which construct above ground nests. **Specialist** indicates species recorded to only collect pollen from one plant family are classified as specialists. **Oil collection** indicates if species is known to collect floral oils. **Overwintering** indicates if a species is known to overwinter as an adult or prepupa. **Origin**, whether the bee is native to the Southwest. **Diurnality**, both nocturnal and crepuscular bees were considered nocturnal. Trait data were inferred for species without sufficient natural history records based on taxonomy.

**Table S1 References:**

| 1. Ackerman, A. J. (1916). The carpenter-bees of the United States of the genus Xylocopa. *Journal of the New York Entomological Society*, *24*(3), 196-232. |
| --- |
| 1. Alcock, J., & Buchmann, S. (2011). The mating system of Habropoda pallida Timberlake (Anthophorinae: Apidae). Journal of insect behavior, 24, 348-362. |
| 1. Armbrust, E. A. (2004). Resource use and nesting behavior of Megachile prosopidis and M. chilopsidis with notes on M. discorhina (Hymenoptera: Megachilidae). *Journal of the Kansas Entomological Society*, *77*(2), 89-98. |
| 1. Bosch, J., Maeta, Y., & Rust, R. (2001). A phylogenetic analysis of nesting behavior in the genus Osmia (Hymenoptera: Megachilidae). *Annals of the Entomological Society of America*, *94*(4), 617-627. |
| 1. Bossert, S., Wood, T. J., Patiny, S., Michez, D., Almeida, E. A., Minckley, R. L., ... & Murray, E. A. (2022). Phylogeny, biogeography and diversification of the mining bee family Andrenidae. Systematic Entomology, 47(2), 283-302. |
| 1. Burks, B. D. (1968). The pollen-collecting bees of the Anthidiini of California (Hymenoptera: Megachilidae). |
| 1. Cane, J. H., & Neff, J. L. (2011). Predicted fates of ground-nesting bees in soil heated by wildfire: thermal tolerances of life stages and a survey of nesting depths. Biological Conservation, 144(11), 2631-2636. |
| 1. Chappell, M. A. (1984). Temperature regulation and energetics of the solitary bee Centris pallida during foraging and intermale mate competition. *Physiological Zoology*, *57*(2), 215-225. |
| 1. Correia, M. (1980). Study of the biology of Heriades truncorum. 1. Biological and morphological aspects. *Apidologie*, *11*(4), 309-339. |
| 1. Cross, E. A., & Bohart, G. E. (1960). The biology of Nomia (Epinomia) triangulifera with comparative notes on other species of Nomia. University of Kansas Science Bulletin, 41(6), 761. |
| 1. Danforth, B. N. (1989). Nesting behavior of four species of Perdita (Hymenoptera: Andrenidae). *Journal of the Kansas Entomological Society*, 59-79. |
| 1. Danforth, B. N., Ji, S., & Ballard, L. J. (2003). Gene flow and population structure in an oligolectic desert bee, Macrotera (Macroteropsis) portalis (Hymenoptera: Andrenidae). *Journal of the Kansas Entomological Society*, 221-235. |
| 1. Danforth, B. N., Minckley, R. L., & Neff, J. L. (2019). The solitary bees: biology, evolution, conservation. Princeton University Press. |
| 1. Diller, S.N., Schaeffer, J.S., Grundel, R., Pavlovic, N.B., McKenna, J.E. Jr., Esselman, P.C., 2020, Bee-Gap: Ecology, Life-History, and Distribution of Bee Species in the United States 2017: U.S. Geological Survey data release, https://doi.org/10.5066/P9QHQNNS. |
| 1. Discoverlife, Ascher J.S., Pickering J. (2017) Discover Life bee species guide and world checklist (Hymenoptera: Apoidea: Anthophila). |
| 1. Dorchin, A., López-Uribe, M. M., Praz, C. J., Griswold, T., & Danforth, B. N. (2018). Phylogeny, new generic-level classification, and historical biogeography of the Eucera complex (Hymenoptera: Apidae). Molecular Phylogenetics and Evolution, 119, 81-92. |
| 1. Dyer, F. C., & Seeley, T. D. (1991). Nesting behavior and the evolution of worker tempo in four honey bee species. *Ecology*, *72*(1), 156-170. |
| 1. Eickwort, G. C. (1981). Aspects of the nesting biology of five Nearctic species of Agapostemon (Hymenoptera: Halictidae). *Journal of the Kansas Entomological Society*, 337-351. |
| 1. Fox, W. J. (1899). Synopsis of the United States species of the hymenopterous genus Centris Fabr. with description of a new species from Trinidad. *Proceedings of the Academy of Natural Sciences of Philadelphia*, *51*(1), 63-70. |
| 1. Frohlich, D. R., & Parker, F. D. (1985). Observations on the nest-building and reproductive behavior of a resin-gathering bee: Dianthidium ulkei (Hymenoptera: Megachilidae). *Annals of the Entomological Society of America*, *78*(6), 804-810. |
| 1. Griswold, T. L. (1986). Notes on the nesting biology of Protosmia (Chelostomopsis) rubifloris (Cockerell). *The Pan-Pacific Entomologist*, *62*, 84. |
| 1. Hurd,Paul D., Jr. and Linsley, E. Gorton. 1975. *The principal Larrea bees of the southwestern United States (Hymenoptera, Apoidea)*. Washington: Smithsonian Institution Press. In *Smithsonian Contributions to Zoology*, 193. https://doi.org/10.5479/si.00810282.193. |
| 1. Krombein, K. V., Hurd, P. D., Smith, D. R., & Burks, B. D. (1979). *Catalog of Hymenoptera in America north of Mexico*(Vol. 1, pp. 1199-2209). Washington, DC: Smithsonian Institution Press. |
| 1. LaBerge, W. E. (1969). A revision of the bees of the genus Andrena of the Western Hemisphere. Part II. Plastandrena, Aporandrena, Charitandrena. *Transactions of the American Entomological Society (1890-)*, *95*(1), 1-47. |
| 1. LaBerge, W. E. (2001). Revision of the bees of the genus Tetraloniella in the New World (Hymenoptera: Apidae). *Illinois Natural History Survey Bulletin; v. 036, no. 03*. |
| 1. Michener, C. D. (1947). A revision of the American species of Hoplitis (Hymenoptera, Megachilidae). Bulletin of the AMNH; v. 89, article 4. |
| 1. Michener, C. D. (1953). The biology of a leafcutter bee (Megachile brevis) and its associates. *University of Kansas Science Bulletin*, *35*(3), 1659. |
| 1. Michener, C. D. (1975). Nests of Paranthidium jugatorium in association with Melitoma taurea (Hymenoptera: Megachilidae and Anthophoridae). *Journal of the Kansas Entomological Society*, 194-200. |
| 1. Michener, C. D., & Bennett, F. D. (1977). Geographical variation in nesting biology and social organization of Halictus ligatus. *The University of Kansas Science Bulletin*, *51*(7), 233. |
| 1. Michener, C. D., & Ordway, E. (1963). The life history of Perdita maculigera maculipennis (Hymenoptera: Andrenidae). *Journal of the Kansas Entomological Society*, *36*(1), 34-45. |
| 1. Miliczky, E. (2008). Observations on the nesting biology of Andrena (Plastandrena) prunorum Cockerell in Washington state (Hymenoptera: Andrenidae). *Journal of the Kansas Entomological Society*, *81*(2), 110-121. |
| 1. Neff, J. L., & Simpson, B. B. (1992). Partial bivoltinism in a ground-nesting bee: the biology of Diadasia rinconis in Texas (Hymenoptera, Anthophoridae). *Journal of the Kansas Entomological Society*, 377-392. |
| 1. Neff, J. L., & Simpson, B. B. (1997). Nesting and foraging behavior of Andrena (Callandrena) rudbeckiae Robertson (Hymenoptera: Apoidea: Andrenidae) in Texas. *Journal of the Kansas Entomological Society*, 100-113. |
| 1. Onuferko, T. M. (2019). A review of the cleptoparasitic bee genus Epeolus Latreille, 1802 (Hymenoptera: Apidae) in the Caribbean, Central America and Mexico. *European Journal of Taxonomy*, (563). |
| 1. Onuferko, T. M., Packer, L., & Genaro, J. A. (2021). Brachymelecta Linsley, 1939, previously the rarest North American bee genus, was described from an aberrant specimen and is the senior synonym for Xeromelecta Linsley, 1939. *European Journal of Taxonomy*, *754*, 1-51. |
| 1. Ordway, E. (1987). The life history of Diadasia rinconis Cockerell (hymenoptera: Anthophoridae). *Journal of the Kansas Entomological Society*, 15-24. |
| 1. Packer, L., Gravel, A. I. D., & Lebuhn, G. (2007). Phenology and social organization of Halictus (Seladonia) tripartitus (Hymenoptera: Halictidae). *Journal of Hymenoptera Research*, *16*, 281-292. |
| 1. Parker, F. D., & Bohart, G. E. (1979). Dolichostelis, a new genus of parasitic bees (Hymenoptera: Megachilidae). *Journal of the Kansas Entomological Society*, 138-153. |
| 1. Parker, F. D., Tepedino, V. J., & Bohart, G. E. (1981). Notes on the biology of a common sunflower bee, Melissodes (Eumelissodes) agilis Cresson. *Journal of the New York Entomological Society*, 43-52. |
| 1. Rehan, S. M., & Richards, M. H. (2010). Nesting biology and subsociality in Ceratina calcarata (Hymenoptera: Apidae). *The Canadian Entomologist*, *142*(1), 65-74. |
| 1. Richards, M. H., & Packer, L. (2010). Social behaviours in solitary bees: interactions among individuals in Xeralictus bicuspidariae Snelling (Hymenoptera: Halictidae: Rophitinae). *J Hym Res*, *19*, 66-76. |
| 1. Rozen Jr, J. G. (1987). Nesting biology of the bee Ashmeadiella holtii and its cleptoparasite, a new species of Stelis (Apoidea: Megachilidae). *American Museum Novitates*, (2900). |
| 1. Rozen Jr, J. G. (1989). Life History Studies of the "Prinmitive" Panurgine Bees (Hymenoptera: Andrenidae: Panurginae). |
| 1. Rozen Jr, J. G. (1992). Biology of the bee Ancylandrena larreae (Andrenidae, Andreninae) and its cleptoparasite Hexepeolus rhodogyne (Anthophoridae, Nomadinae): with a review of egg deposition in the Nomadinae (Hymenoptera, Apoidea). American Museum novitates; no. 3038. |
| 1. Rozen Jr, J. G. (1994). Biologies of the bee genera Ancylandrena (Andrenidae, Andreninae) and Hexepeolus (Apidae, Nomadinae): and phylogenetic relationships of Ancylandrena based on its mature larva (Hymenoptera, Apoidea). American Museum novitates; no. 3108. |
| 1. Rozen Jr, J. G. (2013). Larval development and nesting biology of the adventive wood-nesting bee Lithurgus (L.) chrysurus Fonscolombe (Hymenoptera: Megachilidae: Lithurgini). *American Museum Novitates*, *2013*(3774), 20-40. |
| 1. Rozen Jr, J. G. (2016). The Bee Svastra sabinensis: Nesting Biology, Mature Oocyte, Postdefecating Larva, and Association with Triepeolus penicilliferus (Apidae: Apinae: Eucerini and Nomadinae: Epeolini). *American Museum Novitates*, *2016*(3850), 1-12. |
| 1. Rozen Jr, J. G., & Buchmann, S. L. (1990). Nesting biology and immature stages of the bees Centris caesalpiniae, C. pallida, and the cleptoparasite Ericrocis lata (Hymenoptera, Apoidea, Anthophoridae). American Museum novitates;; no. 2985. |
| 1. Rozen Jr, J. G., & Hall, H. G. (2012). Nesting biology and immatures of the oligolectic bee Trachusa larreae (Apoidea: Megachilidae: Anthidiini). *American Museum Novitates*, *2012*(3765), 1-24. |
| 1. Rozen JR, J. G., & Macneill, C. D. (1957). Biological observations on Exomalopsis (Anthophorula) chionura Cockerell, including a comparison of the biology of Exomalopsis with that of other anthophorid groups (Hymenoptera: Apoidea). *Annals of the Entomological Society of America*, *50*(5), 522-529. |
| 1. Rozen Jr, J. G., & McGinley, R. J. (1976). Biology of the bee genus Conanthalictus (Halictidae, Dufoureinae). American Museum novitates; no. 2602. |
| 1. Rozen Jr, J. G., & McGinley, R. J. (1991). Biology and larvae of the cleptoparasitic bee Townsendiella pulchra and nesting biology of its host Hesperapis larreae (Hymenoptera, Apoidea). American Museum novitates; no. 3005. |
| 1. Rozen Jr, J. G., Roig-Alsina, A., & Alexander, B. A. (1997). The cleptoparasitic bee genus Rhopalolemma: with reference to other Nomadinae (Apidae), and biology of its host Protodufourea (Halictidae, Rophitinae). American Museum novitates; no. 3194. |
| 1. Rust, R. W. (1990). Spatial and temporal heterogeneity of pollen foraging in Osmia lignaria propinqua (Hymenoptera: Megachilidae). *Environmental Entomology*, *19*(2), 332-338. |
| 1. Sakagami, S. F., Hoshikawa, K., & Fukuda, H. (1984). Overwintering ecology of two social halictine bees, Lasioglossum duplex and L. problematicum. *Researches on population ecology*, *26*(2), 363-378. |
| 1. Schwarz, H. F. (1926). *North American Dianthidium, Anthidiellum, and Paranthidium*. American Museum of Natural History. |
| 1. Sheffield, C., Heron, J., & Musetti, L. (2020). Xylocopa sonorina Smith, 1874 from Vancouver, British Columbia, Canada (Hymenoptera: Apidae, Xylocopinae) with comments on its taxonomy. *Biodiversity Data Journal*, *8*. |
| 1. Shinn, A. F. (1967). A revision of the bee genus Calliopsis and the biology and ecology of C. andreniformis (Hymenoptera, Andrenidae). |
| 1. Sommeijer, M. J., Neve, J., & Jacobusse, C. (2012). The typical development cycle of the solitary bee Colletes halophilus. *entomologische berichten*, *72*(1-2), 52-58. |
| 1. Stephen, W. P. (1952). *A revision of the genus Colletes in America North of Mexico (Hymenoptera, Colletidae)* (Doctoral dissertation, University of Kansas). |
| 1. Tepedino, V. J., & Griswold, T. L. (1995). *The bees of the Columbia Basin*. Interior Columbia Basin Ecosystem Management Project. |
| 1. Thorp, R. W. (1969). Ecology and behavior of Anthophora edwardsii (Hymenoptera: Anthophoridae). *American Midland Naturalist*, 321-337. |
| 1. Torchio, P. F. (1984). The nesting biology of Hylaeus bisinuatus Forster and development of its immature forms (Hymenoptera: Colletidae). *Journal of the Kansas Entomological Society*, 276-297. |
| 1. Torchio, P. F., & Trostle, G. E. (1986). Biological notes on Anthophora urbana urbana and its parasite, Xeromelecta californica (Hymenoptera: Anthophoridae), including descriptions of late embryogenesis and hatching. *Annals of the Entomological Society of America*, *79*(3), 434-447. |
| 1. Torchio, P. F., Rozen Jr, J. G., Bohart, G. E., & Favreau, M. S. (1967). Biology of Dufourea and of its cleptoparasite, Neopasites (Hymenoptera: Apoidea). *Journal of the New York Entomological Society*, 132-146. |
| 1. Wcislo, W. T. (1993). Communal nesting in a North American pearly-banded bee, Nomia tetrazonata, with notes on nesting behavior of Dieunomia heteropoda (Hymenoptera: Halictidae: Nomiinae). *Annals of the entomological Society of America*, *86*(6), 813-821. |

**Table S2:** Environmental covariates considered for inclusion in the JSDMs.

| **Abbr.** | **Variable** | ***1971-2000*** | ***2001-2020*** | ***2021 - 2050*** | **Selected** | **Justification** |
| --- | --- | --- | --- | --- | --- | --- |
| **Climate** |  |  |  |  |  |  |
| EXT | Extreme Maximum Temperature^1,2^ | Decade (1971-2000) | Decade (2001-2020) | Decade mean of annual data (2021 - 2100) | ✓ | Extreme heat may contribute to thermal and desiccation stress^3^, and changes to floral community^4^ |
| EMT | Extreme Minimum Temperature^1,2^ | Decade (1971-2000) | Decade (2001-2020) | Decade mean of annual data (2021 - 2100) |  | Warmer winter temperature can increase metabolic demands during overwintering^5^ |
| DD1040 | Degree-days above 10${}^{\circ}$C and below 40${}^{\circ}$C^1,2^ | Decade (1971-2000) | Decade (2001-2020) | Decade mean of annual data (2021 - 2100) |  | Approximates flight period, growing season duration, and may relate to winter mortality^6^ |
| bFFP | Beginning of Frost Free Period^1,2^ | Decade (1971-2000) | Decade (2001-2020) | Decade mean of annual data (2021 - 2100) |  | Possible early boundary for flight period in spring emerging species |
| eFFP | End of Frost Free Period^1,2^ | Decade (1971-2000) | Decade (2001-2020) | Decade mean of annual data (2021 - 2100) |  | Possible late boundary for flight period in late summer/fall emerging species |
| PPT_wt | Winter Precipitation^1,2^ | Decade (1971-2000) | Decade (2001-2020) | Decade mean of annual data (2021 - 2100) | ✓ | Important determinate of bloom^7,8^ |
| PPT_sm | Summer Precipitation^1,2^ | Decade (1971-2000) | Decade (2001-2020) | Decade mean of annual data (2021 - 2100) | ✓ | Important determinate of bloom^8^ |
| AI | Aridity Index^1,2^  $AI=MAP/Eref$ | Decade (1971-2000)  Derived from MAP and Eref | Decade (2001-2020)  Derived from MAP and Eref | Decade mean of annual data (2021 - 2100)  Derived from MAP and Eref |  | Correlated with species richness in global analyses^9^ |
| MAP | Mean Annual Precipitation^1,2^ | Decade (1971-2000) | Decade (2001-2020) | Decade mean of annual data (2021 - 2100) |  | Used in calculation of AI |
| MAT | Mean Annual Temperature^1,2^ | Decade (1971-2000) | Decade (2001-2020) | Decade mean of annual data (2021 - 2100) | ✓ | Warming temperatures may cause thermal stress and higher metabolic demands during overwintering^3,5^ |
| Eref | Hargreaves Reference Evapotranspiration^1,2^ | Decade (1971-2000) | Decade (2001-2020) | Decade mean of annual data (2021 - 2100) |  | Used in calculation of AI |
| **Soils** |  |  |  |  |  |  |
| SSC | Soil sand content at 0cm^10^ | Assumed fixed | Assumed fixed | Assumed fixed | ✓ | Plausible contributor to nest site selection in ground nesting species. May also be a lose proxy for plant community.^11^ |
| SCC | Soil clay content at 0cm^12^ | Assumed fixed | Assumed fixed | Assumed fixed |  | Plausible contributor to nest site selection in ground nesting species. May also be a lose proxy for plant community.^11^ |
| **Topography** |  |  |  |  |  |  |
| Eness | Eastness^13^ | Assumed fixed | Assumed fixed | Assumed fixed | ✓ | Alters nest site suitability and solar exposure^11^ |
| Nness | Northness^13^ | Assumed fixed | Assumed fixed | Assumed fixed | ✓ | Alters nest site suitability and solar exposure^11^ |
| TRI | Terrain Ruggedness Index^13^ | Assumed fixed | Assumed fixed | Assumed fixed | ✓ | Proxy for microclimate availability and habitat heterogeneity^14^ |
| CTI | Compound Topographic Index^13^ | Assumed fixed | Assumed fixed | Assumed fixed |  | Proxy for microclimate availability and habitat heterogeneity^14^ |
| **Land Use** |  |  |  |  |  |  |
| LULC | Land Use, Land Cover^15,16^  (Simplified to forested and unforested cover) | Note: For 1971 - 1990 used data for 1992 | **2010:**  Used ESA^6^ data  **2020:**  Projected data | **All periods and SSPs:**  End points (e.g., for 2020 - 2030 use 2025) data |  | Different land use types support varied floral and bee communities |
| ULF | Urban Land Fraction^17–19^ | Historical Data: 1975 to 20148  Switched to projected data source for 2000 | Ten-year intervals of urban grid cell fraction for 2010 and 2020 | Ten-year intervals of urban grid cell fraction for 2030 to 2050 |  | Urbanization linked to changes in bee diversity and community compositions^20,21^ |
| PA | Protected Areas^22^ | Assumed fixed | Assumed fixed | Assumed fixed | ✓ | Included to facilitate analysis |
| DNA | Distance to Natural Area | Derived from LULC | Derived from LULC | Derived from LULC | ✓ | Urban fringe has more available habitat due to lower density development^23^ |

**Table S2 References:**

1. Wang, T., Hamann, A., Spittlehouse, D. & Carroll, C. Locally downscaled and spatially customizable climate data for historical and future periods for North America. *PLoS ONE* **11**, (2016).

2. Mahony, C. R., Wang, T., Hamann, A. & Cannon, A. J. A global climate model ensemble for downscaled monthly climate normals over North America. *International Journal of Climatology* **42**, 5871–5891 (2022).

3. Johnson, M. G., Glass, J. R., Dillon, M. E. & Harrison, J. F. How will climatic warming affect insect pollinators? in *Advances in Insect Physiology* vol. 64 1–115 (Elsevier, 2023).

4. Moloney, K. A. *et al.* Increased fire risk in Mojave and Sonoran shrublands due to exotic species and extreme rainfall events. *Ecosphere* **10**, e02592 (2019).

5. Williams, C. M., Henry, H. A. L. & Sinclair, B. J. Cold truths: how winter drives responses of terrestrial organisms to climate change. *Biological Reviews* **90**, 214–235 (2015).

6. Sgolastra, F. *et al.* The long summer: Pre-wintering temperatures affect metabolic expenditure and winter survival in a solitary bee. *Journal of Insect Physiology* **57**, 1651–1659 (2011).

7. Beatley, J. C. Phenological Events and Their Environmental Triggers in Mojave Desert Ecosystems. *Ecology* **55**, 856–863 (1974).

8. Bowers, J. E. Has Climatic Warming Altered Spring Flowering Date of Sonoran Desert Shrubs? *The Southwestern Naturalist* **52**, 347–355 (2007).

9. Orr, M. C. *et al.* Global Patterns and Drivers of Bee Distribution. *Current Biology* **31**, 451-458.e4 (2021).

10. Hengl, T. Sand content in % (kg / kg) at 6 standard depths (0, 10, 30, 60, 100 and 200 cm) at 250 m resolution. Zenodo https://doi.org/10.5281/zenodo.2525662 (2018).

11. Antoine, C. M. & Forrest, J. R. K. Nesting habitat of ground-nesting bees: a review. *Ecological Entomology* **46**, 143–159 (2021).

12. Hengl, T. Clay content in % (kg / kg) at 6 standard depths (0, 10, 30, 60, 100 and 200 cm) at 250 m resolution. Zenodo https://doi.org/10.5281/ZENODO.1476854 (2018).

13. Amatulli, G., McInerney, D., Sethi, T., Strobl, P. & Domisch, S. Geomorpho90m, empirical evaluation and accuracy assessment of global high-resolution geomorphometric layers. *Sci Data* **7**, 162 (2020).

14. Title, P. O. & Bemmels, J. B. ENVIREM: an expanded set of bioclimatic and topographic variables increases flexibility and improves performance of ecological niche modeling. *Ecography* **41**, 291–307 (2018).

15. ESA. Land Cover CCI Product User Guide Version 2. Tech. Rep. (2017).

16. Chen, G., Li, X. & Liu, X. Global land projection based on plant functional types with a 1-km resolution under socio-climatic scenarios. *Sci Data* **9**, 125 (2022).

17. Corbane, C., Florczyk, A., Pesaresi, M., Politis, P. & Syrris, V. GHS-BUILT R2018A - GHS built-up grid, derived from Landsat, multitemporal (1975-1990-2000-2014) - OBSOLETE RELEASE. European Commission, Joint Research Centre (JRC) https://doi.org/10.2905/jrc-ghsl-10007 (2018).

18. Gao, J. & Pesaresi, M. Downscaling SSP-consistent global spatial urban land projections from 1/8-degree to 1-km resolution 2000–2100. *Sci Data* **8**, 281 (2021).

19. Gao, J. & Pesaresi, M. Global 1-km Downscaled Urban Land Extent Projection and Base Year Grids by SSP Scenarios, 2000-2100. NASA Socioeconomic Data and Applications Center (SEDAC) (2021).

20. Hostetler, N. E. & McIntyre, M. E. Effects of urban land use on pollinator (Hymenoptera: Apoidea) communities in a desert metropolis. *Basic and Applied Ecology* **2**, 209–218 (2001).

21. Cane, J. H., Minckley, R. L., Kervin, L. J., Roulston, T. H. & Williams, N. M. Complex Responses Within A Desert Bee Guild (Hymenoptera: Apiformes) To Urban Habitat Fragmentation. *Ecological Applications* **16**, 632–644 (2006).

22. USGS GAP. Protected Areas Database of the United States (PAD-US). U.S. Geological Survey data release https://doi.org/10.5066/P9Q9LQ4B (2022).

23. Shrestha, M. K., York, A. M., Boone, C. G. & Zhang, S. Land fragmentation due to rapid urbanization in the Phoenix Metropolitan Area: Analyzing the spatiotemporal patterns and drivers. *Applied Geography* **32**, 522–531 (2012).

**
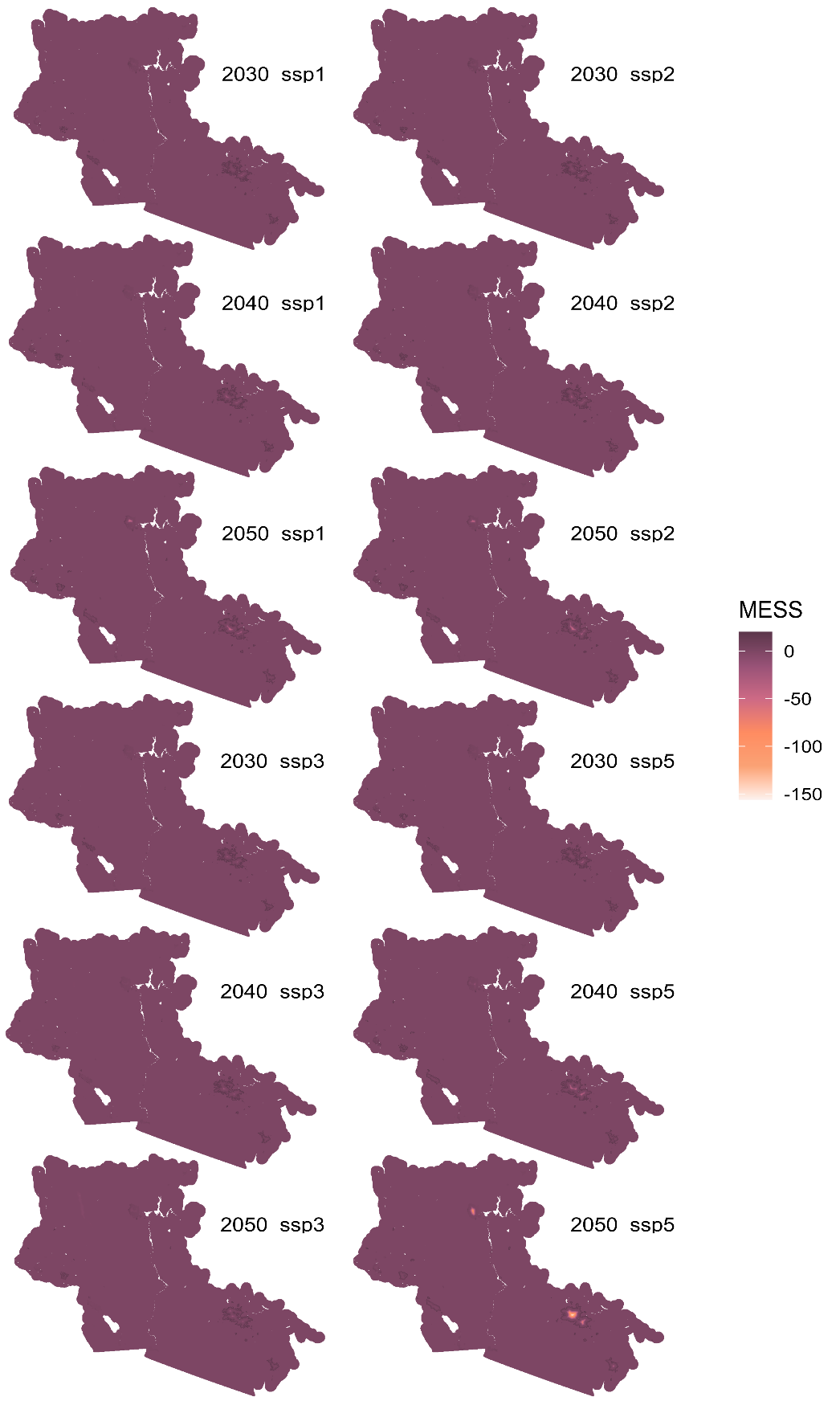
Figure S1:** Multivariate Environmental Similarity Surfaces for each decade and climate change scenarios. Values less than 0 are novel environments compared to the training period. Novel environments are found in the urban cores of major metropolitan areas (>8 km from natural areas).


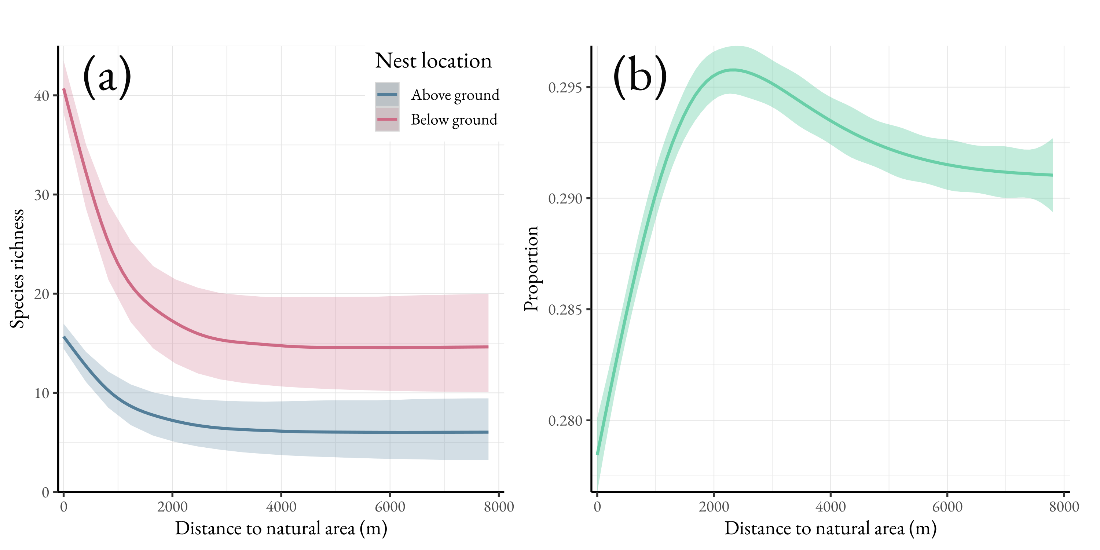


**Figure S2:** The species richness of above and below ground nesting bees across a gradient of distances from natural land uses within urban areas (a). The proportion of above ground nesting bees increases in urban areas (b).
